# Completeness of Classical Thermodynamics: The Ideal Gas, the Unconventional Systems, the Rubber Band, the Paramagnetic Solid and the Kelly Plasma

**DOI:** 10.3390/e22040398

**Published:** 2020-03-31

**Authors:** Karen Arango-Reyes, Gonzalo Ares de Parga

**Affiliations:** Escuela Superior de Física y Matemáticas del Instituto Politécnico Nacional, Edif. 9 U.P. Zacatenco, Ciudad de México 07738, Mexico; neraky_ar@hotmail.com

**Keywords:** fundamental equations, Hessian, entropy, equations of state

## Abstract

A method is developed to complete an incomplete set of equations of state of a thermodynamic system. Once the complete set of equations is found, in order to verify the thermodynamic validity of a system, the Hessian and entropy methods are exposed. An original approach called the completeness method in order to complete all the information about the thermodynamic system is exposed. The Hessian method is improved by developing a procedure to calculate the Hessian when it is not possible to have an expression of the internal energy as a fundamental equation. The entropy method is improved by showing how to prove the first-degree homogeneous property of the entropy without having a fundamental expression of it. The completeness method is developed giving a total study of the thermodynamic system by obtaining the set of independent TdS equations and a recipe to obtain all the thermodynamics identities. In order to show the viability of the methods, they are applied to a typical thermodynamic system as the ideal gas. Some well-known and unknown thermodynamic identities are deduced. We also analyze a set of nonphysical equations of state showing that they can represent a thermodynamic system, but in an unstable manner. The rubber band, the paramagnetic solid and the Kelly equation of state for a plasma are corrected using our methods. In each case, a comparison is made between the three methods, showing that the three of them are complementary to the understanding of a thermodynamic system.

## 1. Introduction

The formulation of classical thermodynamics is normally presented by first explaining the thermodynamic quantities based on physical concepts and giving some examples that permit understanding the three laws of classical thermodynamics. Starting from this, it is possible to define many thermodynamic quantities and to obtain many kinds of relations between them, as for example, Maxwell’s relations. Measurable quantities are defined that allow constructing experimental equations of state to understand the different physical systems [1]. However, many rigorous formulations of classical thermodynamics appeared during the last century probably led by the work done by Caratheodory [2]. These treaties lead us to a deepening of classical thermodynamics. In the early seventies of the last century, Tikody and Hummel [3] proposed a recipe for producing thermodynamic consistent empirical equations of state. However, their analysis was constrained to a particular set of thermodynamic relations without taking into account a more general and complete view. Complementary to this, a very modern formulation done by Callen [4] guides us to perfectly understand the role of the fundamental equations of a thermodynamic system. In fact, one of the more important issues in Callen formulation is the requirement that both entropy and internal energy have to be first-degree homogeneous functions of the extensive variables, S=(U,V,N) for the entropy picture (or entropy representation) and U=(S,V,N) for the internal energy picture (or energy representation). This imposes many restrictions on the equations of state. Moreover, by claiming that ${\left[\partial S/\partial U\right]}_{V,N}$ be positive, the first and the second laws of Thermodynamics are accomplished.

Recently, Essex and Andresen [5] showed that the first-degree homogeneous property of the entropy is equivalent to show that the Hessian possesses an eigenvalue equal to zero and the others have to be positive real numbers. Indeed, they developed this method to check the viability of the empirical equations obtained in some areas of chemistry and engineering.

On the other hand, recently too, it has been shown that for thermodynamic systems with unconventional state equations, the Carnot theorem is valid [6]. Apparently, the Carnot theorem is a universal property for a large number of systems despite not behaving physically. However, such a system may not have a first-degree homogeneous entropy. A method to correct such equations needs to be improved. Our method represents a more general method than the Tikody and Hummel [3] one and a complement for the technique developed by Essex and Andresen [5] to study the equations of state proposed by the experimentalists. Unlike the method of Essex and Andresen, our proposal shows how to correct the equations of state in order to describe a system that complies with thermodynamics. In the process, a technique is developed obtaining a set of thermodynamic identities which describe the system in a complete form. Some of the obtained identities are unknown for each system. Unlike the methods used in [3,4,5], the completeness method aims at providing as much information as possible, namely, by obtaining all the independent TdS relations.

The article is organized as follows. In Section 2, first, we develop a technique for obtaining equations of state which complete a set of equations of state proposed in theoretical or empirical forms that are incomplete in the sense that they are not sufficient in number to describe the system, or possess physical inconsistencies. This is applied in Section 4.2 (Unconventional System: A Particular Case) and Section 4.3 (The Rubber Band). Then, the Hessian method developed by Essex and Andresen [5] is briefly exposed, and it is improved by developing a procedure to calculate the Hessian when it is not possible to explicitly write an expression of the internal energy as a fundamental equation. This is shown in Appendix C (paramagnetic solid case) and Appendix D (Kelly plasma case). The entropy method based on Callen’s postulates [4] is exposed. It represents a technique to test the thermodynamic viability of a set of equations using the first-degree homogeneity of the entropy and the positive characteristic of the partial derivative of the entropy with respect to the internal energy. We develop a technique to demonstrate the first-degree homogeneity of the entropy even without knowing the entropy as a fundamental equation. This is shown in Appendix A, and it is applied in Section 4.5.2 (Entropy method), for the Kelly plasma. In Section 3, the completeness method is exposed. The complete set of TdS equations are obtained in order to obtain the independent TdS equations which generate all the others. Starting from such equations, thermodynamic identities which give a complete description of a thermodynamic system are derived. In Section 4, we first apply the three methods to the ideal gas with *N* variable, obtaining the regular results and showing some novel identities between the different heat capacities, as for example Equations (29) and (31). A set of nonphysical equations of state is analyzed giving a well-defined but unstable thermodynamic system. Additionally, a novel identity is obtained between the heat capacities, Equation (46). Using the entropy method, the rubber band equations of state that appear in the literature are corrected by considering the number of monomers in order to obtain the set of equations of state that describes the thermodynamic system. The paramagnetic solid with *N* variable is analyzed showing the changes that must be done in the expression of the TdS equations in order to obtain a real thermodynamic system. Finally, we deduce the complementary equation of state that has to accompany the Kelly equation of a plasma [7], in order to obtain a thermodynamic system. Some concluding remarks are made in Section 5.

## 2. The Constraints of the Equations of State in Classical Thermodynamics: Two Methods

A set of equations of state may not represent a thermodynamic system. In order to test if such a set accomplishes the laws of classical thermodynamics, it is necessary to verify some properties. First of all, it has to be determined if the number of equations entirely described the thermodynamic system. Obviously, the independence of each equation has to be analyzed, and secondly, the number of equations must be equal to the degree of freedom of the system. Two different methods are set out to ensure that the set of equations represents a thermodynamic system. There are some properties that are common to both methods, and they must be described.

### 2.1. Completing the Number of Equations

As was mentioned in the introduction, empirical state equations coming from experimentalists in some areas of physics, chemistry and engineering are in many cases incomplete in the sense that they are not sufficient in number to describe the system; that is, for example, we can deal with a system with two degrees of freedom (n=2), and we know just one equation of state. We need to propose another equation of state in order to obtain a complete set of equations. In general, this is done by identifying the intensive and extensive variables and then by using the equations of state in the equation of the internal energy. Considering the fact that internal energy is a state function, the second partial derivatives of the internal energy must be equal to each other by interchanging the order of the partial derivatives (∂U/∂x∂y=∂U/∂y∂x). Hence, it is possible to propose another equation of state to complete the system. In such a way, we obtain a complete set of equations of state for the system with the purpose of applying the below methods to check the thermodynamic viability of the system. We apply this method in the examples entitled “The Unconventional System: a Particular Case”, the “Rubber Band”, and in the “Kelly Equation for a Plasma” in Section 4.

### 2.2. The Calculation of the Entropy

It has to be highlighted that to have a complete knowledge of a system, it is necessary to know the chemical potential. In some cases, the entropy of a system is not obtained correctly if the variation of the number of particles *N* s not included. For example, if the entropy of the ideal gas is calculated without permitting the variation of the *N* particles, it will not comply with the first-degree homogeneous property (see, for example, [1]). As we mentioned before, the fact of including the calculation of the chemical potential (Gibbs–Duhem method) gives the correct Boltzmann counting because considering the term μdN (μ the chemical potential) in the first law implies the indistinguishability of the particles. In particular, in “The Rubber Band” in Section 4, we consider the variation of the monomers in order to obtain the chemical potential and, consequently, the correct entropy.

### 2.3. The Third Law of Thermodynamics

Although the third law of Thermodynamics must be satisfied, it is a well-known fact that the equations of state such as for the ideal gas, for a plasma at high temperature or empirical equations of state do not conform to this property. Therefore, we do not ask for compliance with the third law. However, since the entropy must be a monotonically increasing function with respect to the internal energy and when the temperature tends to zero, its limit is zero, we must ask the entropy to be positive, that is:

(1) S≥0.

### 2.4. Two Methods

Once we have a complete set of equations, we have to test the viability of it. We expose two methods that can be used for this purpose. Nevertheless, as we see, it is not always possible to use them directly, and it is necessary to develop some techniques for their applications. For example, sometimes it is not possible to have an analytical expression for the internal energy as a function of the entropy, the volume, and the number of particles, and consequently, the second-order partial derivatives needed to calculate the Hessian have to be derived indirectly.

#### 2.4.1. Hessian Method

From the third postulate of Callen’s formulation [4], it can be deduced that the fundamental equations, S=S(U,V,N) in the entropy picture or U=U(S,V,N) in the energy picture, must be first-degree homogeneous functions, that is:

(2) $$S_{\lambda }=S\left(\lambda U,\lambda V,\lambda N,\ldots \right)=\lambda S\left(U,V,N,\ldots \right),$$

or equivalently

(3) $$U_{\lambda }=U\left(\lambda S,\lambda V,\lambda N,\ldots \right)=\lambda U\left(S,V,N,\ldots \right).$$

Essex and Andersen [5] showed that the Hessian $H=U_{ij}$ ($U_{ij}=\frac{\partial U}{\partial X_{i}\partial X_{j}}$ with $U(X^{0},X^{1},\ldots ,X^{n})$ the internal energy, $S=X^{0}$ and $X^{1},\ldots ,X^{n}$ the corresponding extensive variables) must have a null eigenvalue and the other eigenvalues must be positive real in order for the second law to be respected. Therefore, it is required that from a set of equations of state that tries to describe a thermodynamic system, the internal energy satisfying such property can be derived without requiring to demonstrate the first-degree homogeneous property; It has to be noted that the internal energy possesses n+1 degrees of freedom, that is, $U=U(X^{0},X^{1},X^{2},\ldots ,X^{n})$. Consequently, the Hessian $U_{ij}=\partial^{2}U/\partial X_{i}\partial X_{j}$ is a $\left(n+1\right)\times \left(n+1\right)$ matrix. Finally, Essex and Andersen [5] proposed this method to check the viability of the equations of state given by the experimentalists. However, this method presents three drawbacks. The first is that the internal energy cannot always be expressed algebraically as a function of entropy and its extensive variables, greatly complicating the calculation of the Hessian. The second inconvenience is that in many cases, experimentalists give a system of incomplete state equations (this point is corrected by applying the method described in Section 2.1). These cases appear in the examples that we analyze and solve. The third consists of noticing that Essex and Andresen’s method [5] requires calculating all the eigenvalues in order to know the relaxation times that describe the behavior of the disturbances. In reality, to check the viability of the equations of state, it is just necessary to demonstrate the existence of a null eigenvalue because once we obtain the symmetrical Hessian ($U_{ij}=\partial^{2}U/\partial X_{i}\partial X_{j}=\partial^{2}U/\partial X_{j}\partial X_{i}=U_{ji}$), it is known that the other eigenvalues must be real positive. In reality, to check that a function represents a state function, it is sufficient to be twice differentiable (Schwarz’s theorem or Clairaut’s theorem). That is, it is just necessary to check the existence of $\partial S/\partial X^{i}\partial X^{j}$ or $\partial U/\partial X^{i}\partial X^{j}$, without taking into account the way of differentiating the second partial derivative (interchanging the sequence of differentiation).

##### Recipe of the Hessian Method

To test the viability of a set of equations, the following properties must be satisfied:

**A**  Complete the set of equations
**B**  Calculate the internal energy as a fundamental equation, that is:
  (4) $$U=U(S,V,N,X^{k}),$$
  where $X^{k}$ represents any set of extensive variables of the thermodynamic system.
**C**  Calculate the Hessian. If it is not possible to obtain an analytical expression for the internal energy *U*, the Hessian must be calculated by means of using the thermodynamic identities (Maxwell’s relations, for example) as it is described in Section 4 (for example, the Paramagnetic Solid and the Kelly Plasma).
**D**  Show that the Hessian possesses a null eigenvalue (λ=0).
**E**  Although it is not necessary to demonstrate that the other eigenvalues are positive, calculating them gives more information on the system in order to relate them with its relaxation time.

It has to be highlighted that in this method, it is not necessary to check that the internal energy or the entropy are first-degree homogeneous functions and that the temperature is positive, since both properties are satisfied if items **A**, **C**, and **D** are fulfilled, because they are equivalent to the second law of classical thermodynamics [5]. In addition, this method can be used when the entropy cannot be expressed analytically as a fundamental equation because, if possible, it would be easier to use the entropy method that we describe below.

#### 2.4.2. Entropy Method

As we mentioned before, from the third postulate of Callen’s formulation [4], the entropy method is the simplest one to check the viability of a set of equations because it is only necessary to check that the entropy is positive, that the entropy or the internal energy are first-degree homogeneous functions, and that their derivatives of one with respect to the other are positive.

##### Recipe of the Entropy Method

To test the viability of a set of equations, the following properties must be satisfied:

**A**  Complete the set of equations
**F**  Calculate the entropy as a fundamental equation, that is:
  (5) $$S=S(U,X^{1},\ldots ,X^{n}),$$
  where $X^{k}$ belongs to the set of extensive variables of the thermodynamic system.
**G**  Prove that Equation (1) is satisfied (S≥0) and that the entropy represents a state function. It is sufficient to be twice differentiable (Schwarz’s theorem or Clairaut’s theorem). That is, it is just necessary to check the existence of $\partial S/\partial X^{i}\partial X^{j}$ without taking into account the sequence of differentiation of the second partial derivatives.
**H**  The temperature must be a positive quantity:
  (6) $${\left[\frac{\partial S}{\partial U}\right]}_{V,N}>0\;\mathrm{or}\;{\left[\frac{\partial U}{\partial S}\right]}_{V,N}>0.$$
**I**  Prove that the entropy is a first-degree homogeneous function, that is, Equation (2) must be satisfied.

When it is not possible to write explicitly the entropy as a fundamental equation, a method is developed in Appendix A to test the validity of item **I**.

## 3. Completeness Method of a Thermodynamic System

Once one of the two methods described above has been used to test the viability of a set of equations, it is necessary to analyze the system. Although when we know either of the fundamental equations ($S=S(U,X^{1},\ldots ,X^{n})$ or $U=U(S,X^{1},\ldots ,X^{n})$), it is supposed that all the properties of the system are known, it is necessary to know all the TdS equations and all the possible thermodynamic identities of the system. The method that is to be developed is based on the TdS equations. Therefore, it is necessary to analyze the degree of freedom and the different representations of the system. The degree of freedom is described in the fundamental equation of state and in the general case by n+1 extensive variables as:

(7) $$U=U(X^{0},X^{1},\ldots ,X^{n}),$$

where $X^{0}=S$ and the other *n* extensive variables $X^{j}$ (with j≠0) may be represented by the volume *V*, the number of particles *N*, the magnetization, etc. The equations are derived using the following relations:

(8) $$\left[\frac{\partial U}{\partial X^{j}}\right]=x_{j}\;\mathrm{with}\;j=0,1,2,\ldots n$$

where $x_{j}$ represents the corresponding intensive variables (Gibb’s formula). We need to correctlydescribe Equation (8). For example,

(9) $$\begin{array}{ccc} {\left[\frac{\partial U}{\partial X^{0}}\right]}_{\left\{X^{n};-X^{0}\right\}} & = & {\left[\frac{\partial U}{\partial S}\right]}_{\left\{X^{n};-S\right\}}=T\left(X^{0},X^{1},\ldots ,X^{n}\right)\\ & & \mathrm{and}\\ {\left[\frac{\partial U}{\partial X^{1}}\right]}_{\left\{X^{n};-X^{1}\right\}} & = & {\left[\frac{\partial U}{\partial V}\right]}_{\left\{X^{n};-V\right\}}=-P\left(X^{0},X^{1},\ldots ,X^{n}\right), \end{array}$$

where the set $\left\{X^{n};-X^{l}\right\}$ represents the set composed by all the extensive variables $\left\{X^{n}\right\}$ without $X^{l}$. From these equations of state, sometimes it is possible to analytically express the entropy and the other extensive variables as functions of the intensive and extensive variables

(10) $$U=U(\left\{x_{i}\right\},\left\{X^{j}\right\}),$$

where $\left\{x_{i}\right\}$ represents any set of *i* intensive variables chosen from the set of n+1 possible intensive variables and $\left\{X^{j}\right\}$ any set of *j* extensive variables of the set of *n* extensive variables without the entropy such that the number of total variables is $\left(i+j\right)=\left(n+1\right)$, for example, U=U(T,V,N) for the ideal gas. The functionality of *U* describes the number of representations depending on the form of the equations, the possible different sets $\left\{\left\{x_{i}\right\},\left\{X^{j}\right\}\right\}$ being all the possible representations of the thermodynamic system. It has to be highlighted that some equations of state reduce the number of representations as in the case of the photon gas. These cases are not analyzed in this work since the consequences consist of reducing the degree of freedom. It is important to note that when proposing the equations, the experimentalists choose the representation of the system and, from these equations, the fundamental equations can be derived.

### 3.1. The TdS Equations

The first law of thermodynamics is δQ=dU+δW, where *W* represents the total work done on the system, which in the general case must be written as

(11) $$TdS=dU-x_{i}dX^{i},$$

Gibb’s formula and note that $V=X^{1}$ and the pressure $P=-x_{1}=-\delta U/\delta V$), where the Einstein convention has been assumed for the *i* summation. It has to be noted that we cannot propose the representation $U=U(X^{0},X^{1},\ldots ,X^{n})$ because, were it to be used, Equation (11) would turn to

(12) $$TdX^{0}=TdX^{0}+x_{i}dX^{i}-x_{i}dX^{i},$$

which of course does not give any information. Moreover, it is easy to see that including any representation where $X^{0}$ appears (the entropy), $U=U(\left\{x_{i}\right\};X^{0};\left\{X^{j}\right\})$ (in this case, i+j=n) will not give any information when we try to use a TdS equation. Therefore, in order to obtain the TdS equations, we must consider representations where the entropy is excluded, that is $U=U(\left\{x_{i}\right\},\left\{X^{j}\right\})$ with $X^{0}\notin \left\{X^{j}\right\}$.

Therefore, to build a TdS equation, we need a representation of the following form

(13) $$U=U\left(\left\{x^{i}\right\};\left\{X^{j}\right\}\right)\;\mathrm{with}\;i+j=n+1\;\mathrm{and}\;X^{0}\notin \left\{X^{j}\right\}.$$

### 3.2. The Complete Representation:

The TdS equations take all the following forms by making all the possible representations $\left(\left\{x_{i}\right\},\left\{X^{j}\right\}\right)$ and can be written as

(14) $$\begin{array}{cc} & TdX^{0}\\ = & \left[{\left[\frac{\partial U}{\partial x_{\alpha }}\right]}_{\left\{x_{i};-x_{\alpha }\right\},\left\{X^{j}\right\}}-x_{\lambda }{\left[\frac{\partial X^{\lambda }}{\partial x_{\alpha }}\right]}_{\left\{x_{i};-x_{\alpha }\right\}\left\{X^{j}\right\}}\right]dx^{\alpha }\\ & +\left[{\left[\frac{\partial U}{\partial X^{\alpha }}\right]}_{\left\{x_{i}\right\},\left\{X^{j};-X^{\alpha }\right\}}-x_{\alpha }-x_{\lambda }{\left[\frac{\partial X^{\lambda }}{\partial X^{\alpha }}\right]}_{\left\{x_{i}\right\}\left\{X^{j};-X^{\alpha }\right\}}\right]dX^{\alpha }. \end{array}$$

All the TdS equations can be expressed as a row of a vector identity as follows:

(15) $$\left(\begin{array}{c} TdS\\ TdS\\ \vdots \\ \vdots \\ \vdots \\ \overset{\cdot }{:}\\ \overset{\cdot }{:}\\ \overset{\cdot }{:}\\ \overset{\cdot }{:}\\ \overset{\cdot }{:}\\ \overset{\cdot }{:}\\ \overset{\cdot }{:}\\ \overset{\cdot }{:}\\ \overset{\cdot }{:}\\ \overset{\cdot }{:}\\ \overset{\cdot }{:}\\ \overset{\cdot }{:}\\ \overset{\cdot }{:} \end{array}\right)=\left(\begin{array}{cccccccccc} a_{0}^{0} & a_{1}^{0} & \vdots & a_{n-1}^{0} & a_{n}^{0} & 0 & 0 & \vdots \; & \ldots & 0\\ a_{0}^{1} & a_{1}^{1} & \vdots & a_{n-1}^{1} & 0 & A_{1}^{1} & 0 & \vdots \; & \ldots & 0\\ a_{0}^{2} & a_{1}^{2} & \vdots & a_{n-1}^{2} & 0 & 0 & A_{2}^{2} & \vdots \; & \ldots & 0\\ \overset{\cdot }{:} & \overset{\cdot }{:} & \overset{\cdot }{:} & \overset{\cdot }{:} & \overset{\cdot }{:} & \overset{\cdot }{:} & \overset{\cdot }{:}\; & \overset{\cdot }{:} & \overset{\cdot }{:} & \overset{\cdot }{:}\\ a_{0}^{n-1} & \overset{\cdot }{:} & \overset{\cdot }{:} & a_{n-1}^{n-1} & 0\; & \overset{\cdot }{:} & \overset{\cdot }{:} & \overset{\cdot }{:} & A_{n-1}^{n-1} & 0\\ a_{0}^{n} & a_{1}^{n} & \vdots & a_{n-1}^{n} & 0 & 0 & 0 & \vdots & 0 & A_{n}^{n}\\ a_{0}^{n+1} & a_{1}^{n+1} & \vdots & 0 & a_{n}^{n+1} & A_{1}^{n+1} & 0 & \vdots & 0 & 0\\ a_{0}^{n+2} & a_{1}^{n+2} & \vdots & 0 & a_{n}^{n+2} & 0 & A_{2}^{n+2} & \vdots & 0 & 0\\ a_{0}^{n+3} & \ldots & \vdots & 0 & a_{n}^{n+3} & 0 & 0 & \vdots & \ldots & 0\\ \overset{\cdot }{:} & \overset{\cdot }{:} & \overset{\cdot }{:} & \overset{\cdot }{:} & \overset{\cdot }{:} & \overset{\cdot }{:} & \overset{\cdot }{:}\; & \overset{\cdot }{:} & \overset{\cdot }{:} & \overset{\cdot }{:}\\ a_{0}^{2n} & a_{1}^{2n} & \vdots & 0 & a_{n}^{2n} & 0 & \vdots & \vdots & 0 & A_{n}^{2n}\\ \overset{\cdot }{:} & \overset{\cdot }{:} & \overset{\cdot }{:} & \overset{\cdot }{:} & \overset{\cdot }{:} & \overset{\cdot }{:} & \overset{\cdot }{:}\; & \overset{\cdot }{:} & \overset{\cdot }{:} & \overset{\cdot }{:}\\ \overset{\cdot }{:} & \overset{\cdot }{:} & \overset{\cdot }{:} & \overset{\cdot }{:} & \overset{\cdot }{:} & \overset{\cdot }{:} & \overset{\cdot }{:}\; & \overset{\cdot }{:} & \overset{\cdot }{:} & \overset{\cdot }{:}\\ \overset{\cdot }{:} & \overset{\cdot }{:} & \overset{\cdot }{:} & \overset{\cdot }{:} & \overset{\cdot }{:} & \overset{\cdot }{:} & \overset{\cdot }{:}\; & \overset{\cdot }{:} & \overset{\cdot }{:} & \overset{\cdot }{:}\\ \overset{\cdot }{:} & \overset{\cdot }{:} & \overset{\cdot }{:} & \overset{\cdot }{:} & \overset{\cdot }{:} & \overset{\cdot }{:} & \overset{\cdot }{:}\; & \overset{\cdot }{:} & \overset{\cdot }{:} & \overset{\cdot }{:}\\ \overset{\cdot }{:} & \overset{\cdot }{:} & \overset{\cdot }{:} & \overset{\cdot }{:} & \overset{\cdot }{:} & \overset{\cdot }{:} & \overset{\cdot }{:}\; & \overset{\cdot }{:} & \overset{\cdot }{:} & \overset{\cdot }{:}\\ 0 & 0 & \vdots & 0 & a_{n}^{\left(\frac{\left(2n+1\right)!}{(n+1)!n!}\right)} & A_{1}^{\left(\frac{\left(2n+1\right)!}{(n+1)!n!}\right)} & \vdots & \vdots & \vdots & A_{n}^{\left(\frac{\left(2n+1\right)!}{(n+1)!n!}\right)} \end{array}\right)\left(\begin{array}{c} dx_{0}\\ dx_{1}\\ \ldots \\ dx_{n-1}\\ dx_{n}\\ dX_{1}\\ dX_{2}\\ \ldots \\ dX_{n-1}\\ dX_{n} \end{array}\right)$$

where

(16) aαk=∂U∂xαxi;−xα,Xj−xλ∂Xλ∂xαxi;−xαXj  andAαk=∂U∂Xαxi,Xj;−Xα−xα−xλ∂Xλ∂XαxiXj;−Xα

where the *k*-th entry corresponds to a certain representation $\left\{\left\{x_{i}\right\}\left\{X^{j}\right\}\right\}=\left\{k\right\}$ following the order imposed in Equation (15), and the TdS equation is denoted as the *k*-equation.

### 3.3. The Independent TdS Equations

This gives $\left(\frac{\left(2n+1\right)!}{(n+1)!n!}\right)$TdS equations, but they are not independent. Indeed, in the general case, we just have n+1 independent TdS equations. For example, If we take the 0- and the 1- equations, we can generate the $\left(n^{2}+1\right)$-equation; that is:

(17) $$\begin{array}{ccc} TdS & = & a_{0}^{0}dx_{0}+\ldots +a_{k}^{0}dx_{k}+\ldots +a_{n}^{0}dx_{n}\\ TdS & = & a_{0}^{1}dx_{0}+\ldots +a_{n-1}^{1}dx_{n-1}+A_{1}^{1}dX^{1} \end{array}$$

Then, multiplying the 0-equation by $a_{0}^{1}$ and the 1-equation by $a_{0}^{0}$, and subtracting one from the other, we arrive to

(18) $$\begin{array}{ccc} TdS & = & \frac{\left(a_{0}^{1}a_{1}^{0}-a_{0}^{0}a_{1}^{1}\right)}{\left(a_{0}^{1}-a_{0}^{0}\right)}dx_{1}+\ldots +\frac{\left(a_{0}^{1}a_{k}^{0}-a_{0}^{0}a_{k}^{1}\right)}{\left(a_{0}^{1}-a_{0}^{0}\right)}dx_{k}+\ldots \\ & & +\frac{\left(a_{0}^{1}a_{n-1}^{0}-a_{0}^{0}a_{n-1}^{1}\right)}{\left(a_{0}^{1}-a_{0}^{0}\right)}dx_{n-1}+\frac{a_{0}^{1}a_{n}^{0}}{\left(a_{0}^{1}-a_{0}^{0}\right)}dx_{n}-\frac{a_{0}^{0}A_{1}^{1}}{\left(a_{0}^{1}-a_{0}^{0}\right)}dX^{1}. \end{array}$$

That is, we find the $\left(n^{2}+1\right)$-equation and accordingly, we obtain

(19) $$\begin{array}{ccc} \frac{\left(a_{0}^{1}a_{1}^{0}-a_{0}^{0}a_{1}^{1}\right)}{\left(a_{0}^{1}-a_{0}^{0}\right)} & = & a_{1}^{n^{2}+1},\;\frac{\left(a_{0}^{1}a_{k}^{0}-a_{0}^{0}a_{k}^{1}\right)}{\left(a_{0}^{1}-a_{0}^{0}\right)}=a_{k}^{n^{2}+1}\\ \frac{a_{0}^{1}a_{n}^{0}}{\left(a_{0}^{1}-a_{0}^{0}\right)} & = & a_{n}^{n^{2}+1}\;\mathrm{and}\;-\frac{a_{0}^{0}A_{1}^{1}}{\left(a_{0}^{1}-a_{0}^{0}\right)}=A_{1}^{n^{2}+1}. \end{array}$$

With this technique, we can generate all the other TdS equations, starting from the first $\left(n+1\right)$ equations. Equation (19) represents the technique in order to obtain some thermodynamic identities, and it is used in the examples for obtaining novel relations between the thermodynamic quantities. However, these thermodynamic identities do not always give different or novel relations, so we cannot count how many can be obtained.

On the other hand, it has to be highlighted that it is not necessary to use the first (n+1)TdS equations. Indeed, for a complete representation of the TdS equations, it is necessary just to use n+1 independent TdS equations from Equation (15). Of course, the set of all the differentials must contain all the different differentials of the set ($dx_{i},dX^{j}$). Let us propose a set of (n+1) independent TdS equations sufficient to generate all the other TdS equations. That is, from all the distinct representations, we just need to use n+1 of them which involve all the differentials of the intensive and extensive variables. This implies that it is sufficient to reduce Equation (15) to a simpler matrix identity by choosing the lines which represent the n+1 independent representations. For example, if we choose the first (n+1)TdS equations, the matrix representation turns to:

(20) $$\left(\begin{array}{c} TdS\\ \overset{\cdot }{:}\\ \overset{\cdot }{:}\\ \overset{\cdot }{:}\\ \overset{\cdot }{:}\\ TdS \end{array}\right)=\left(\begin{array}{cccccccccc} a_{0}^{0} & a_{1}^{0} & \ldots & a_{n-1}^{0} & a_{n}^{0} & 0 & 0 & 0 & \ldots & 0\\ a_{0}^{1} & a_{1}^{1} & \ldots & a_{n-1}^{1} & 0 & A_{1}^{1} & 0 & 0 & \ldots & 0\\ a_{0}^{2} & a_{1}^{2} & \ldots & a_{n-1}^{2} & 0 & 0 & A_{2}^{2} & 0 & \ldots & 0\\ \overset{\cdot }{:} & \overset{\cdot }{:} & \overset{\cdot }{:} & \overset{\cdot }{:} & \overset{\cdot }{:} & \overset{\cdot }{:} & \overset{\cdot }{:}\; & \overset{\cdot }{:} & \overset{\cdot }{:} & \overset{\cdot }{:}\\ a_{0}^{n-1} & \overset{\cdot }{:} & \overset{\cdot }{:} & a_{n-1}^{n-1} & 0\; & \overset{\cdot }{:} & \overset{\cdot }{:} & \overset{\cdot }{:} & A_{n-1}^{n} & 0\\ a_{0}^{n} & a_{1}^{n} & \ldots & a_{n-1}^{n} & 0 & 0 & 0 & \ldots & 0 & A_{n}^{n} \end{array}\right)\left(\begin{array}{c} dx_{0}\\ dx_{1}\\ \ldots \\ dx_{n-1}\\ dx_{n}\\ dX_{1}\\ dX_{2}\\ \ldots \\ dX_{n-1}\\ dX_{n} \end{array}\right)$$

As we mentioned in the Introduction, unlike the methods used in [3,4,5], the completeness method aims at providing as much information as possible, namely, by obtaining all the independent TdS relations and giving new thermodynamic identities that are not obtained by using the other methods.

## 4. Examples

In order to apply the methods described in Section 2 and Section 3, some examples are analyzed. First, we start with the ideal gas with *N* variable. All the classical results are recovered for this case. The completeness method permits obtaining certain novel identities. Then, an unconventional equation of state is studied and procuring the other equation of state in order to get a thermodynamic system. The rubber band with variable length is described giving a correct thermodynamic set of equations. Starting with the correct expression of the entropy, the paramagnetic solid system is exposed in order to find the correct expressions of the TdS equations. Finally, a equation proposed by Kelly [7] is analyzed, adding a equation in order to find a complete thermodynamic system.

### 4.1. Ideal Gas with *N* Variable

Let us analyze the ideal gas with *N* variable as is required in order to describe the thermodynamic system in a complete form.

For the ideal gas, the well-known equations of state are

(21) $$PV=NkT\;\mathrm{and}\;U=\frac{3}{2}NkT.$$

From this pair of equations of state, we can deduce all the properties of the ideal gas with the *N* variable. Note that calculating the entropy of the ideal gas without considering *N* as a variable leads to an expression of the entropy which does not comply with the first-degree homogeneous condition. Including the chemical potential corrects the expression of the entropy.

#### 4.1.1. Calculation of Entropy with *N* Variable

Using Equation (21), by integrating the the Gibbs–Duhem relation in order to deduce the chemical potential μ and substituting the three equations of state into the Euler equation, we have

(22) $$S=Ns_{0}+Nk\ln\left[{\left(\frac{U}{U_{0}}\right)}^{\frac{3}{2}}\left(\frac{V}{V_{0}}\right){\left(\frac{N}{N_{0}}\right)}^{-\frac{5}{2}}\right].$$

with $s_{0}=\frac{5}{2}k-N{\left(\frac{\mu }{T}\right)}_{0}$ and μ the chemical potential. The entropy *S* is homogeneous of first degree. Note that with this method, using the chemical potential, the first-degree homogeneous property is achieved without using Boltzmann counting. This is because when the number of particles *N* is considered as variable, the particles are considered as indistinguishable. This result has not been noted before. In reality, in order to assure that the entropy is positive, it is required to know via experiments the value of the constant in order to be able to evaluate the chemical potential μ. However, the entropy can be expressed using statistical mechanics and obtaining the Sackur–Tetrode equation, evaluating all the constants in Equation (22), and observing that the entropy S>0. Nevertheless, to be consistent with the method, we have elected to keep Equation (22).

On the other hand, we can explicitly write the expression of the internal energy from the expression of the entropy, Equation (22).

(23) $$U=U_{0}{\left(\frac{V}{V_{0}}\right)}^{-\frac{2}{3}}{\left(\frac{N}{N_{0}}\right)}^{\frac{5}{3}}e^{\frac{2S-2Ns_{0}}{3Nk}}.$$

#### 4.1.2. Hessian Method

The characteristic polynomial of the Hessian is

$$\begin{array}{c} \left(-6N^{6}k^{2}V^{2}-6N^{4}k^{2}V^{4}-6N^{4}k^{2}V^{2}S^{2}\right)\lambda \\ +\left(5N^{4}k^{2}+5N^{2}k^{2}V^{2}-4NkV^{2}S+2V^{2}S^{2}+2N^{2}V^{2}\right)\lambda^{2}-\lambda^{3}=0. \end{array}$$

It is easy to see that we obtain three different eigenvalues, which are:

$$\lambda =0\;and\;\lambda_{\pm }=\alpha \pm \beta$$

where

$$\alpha =\left[N^{2}+\frac{3}{5}S^{2}+\frac{5}{2}N^{2}{\left(k-\frac{2}{5}\frac{S}{N}\right)}^{2}\right]V^{2}+\frac{5}{2}N^{4}k^{2}$$

and

$$\beta =\frac{1}{2}{\left[\begin{array}{c} -16V^{4}N^{3}Sk-4k^{2}V^{2}N^{4}S^{2}-40k^{3}V^{2}N^{5}S-40V^{4}N^{3}k^{3}S\\ -4k^{2}N^{6}V^{2}-4V^{4}N^{4}k^{2}+8V^{4}N^{2}S^{2}+50k^{4}V^{2}N^{6}-16V^{4}S^{3}kN\\ +25V^{4}N^{4}k^{4}+4V^{4}N^{4}+25N^{8}k^{4}+4V^{4}S^{4}+36V^{4}N^{2}k^{2}S^{2} \end{array}\right]}^{\frac{1}{2}}.$$

This result coincides with that of Essex and Andresen [5], and it proves that the ideal gas described by the equations of state, Equation (21), represents a thermodynamic system.

#### 4.1.3. Entropy Method

We have already proven items **A**, **F**, **G**, and **I**. We just need to prove that item **H** must be satisfied, that is:

$${\left[\frac{\partial U}{\partial S}\right]}_{V,N}=\frac{2}{3Nk}BV^{-\frac{2}{3}}N^{\frac{5}{3}}e^{\frac{2S}{3Nk}}=T>0.$$

where $B=U_{0}V_{0}^{\frac{2}{3}}N_{0}^{-\frac{5}{3}}e^{\frac{-2s_{0}}{3k}}$. The entropy method also shows that the set of equations, Equation (21), represents a thermodynamic system.

#### 4.1.4. Completeness Method

To exemplify our method, let us analyze the TdS equations. All the TdS equations can be expressed using Equations (15) and (16). In this case, we obtain 10 TdS equations; however, as we note in Section 3, only three independent ones are necessary to generate the other TdS equations and to obtain all the information about the thermodynamic system. Let us choose the 2-, 3-, 4-, and 9-equations (see Appendix B for the derivations of such equations).

The 2-equation is:

(24) $$TdS=\frac{5}{2}NkdT-\frac{NkT}{P}dP+\left[\frac{5}{2}kT-\mu \right]dN.$$

Note that this equation does not coincide with any of the three TdS equations normally reported in the literature [8]. This is because normally, the ideal gas is analyzed considering that *N* is fixed, and consequently, we have two variables.

The 3-equation is:

(25) $$TdS=N\left[\frac{15}{2}k-\frac{3\mu }{T}+\frac{\mu^{2}}{kT^{2}}\right]dT+N\left[\frac{3}{2}-\frac{\mu }{kT}\right]d\mu +\frac{N}{V}\left[\frac{5}{2}kT-\mu \right]dV.$$

The 4-equation is:

(26) $$TdS=\frac{N\mu }{T}dT-Nd\mu +\left[\frac{5}{2}kT-\mu \right]dN.$$

##### Thermodynamic Identities

With the three independent TdS equations, it is possible to apply the method described in Section 3 by multiplying and subtracting each from each other and obtain a new TdS equation. Moreover, in this way, we can obtain different thermodynamic identities. Note that using Equation (14), we can define in this case where i≤n

(27) $$\begin{array}{ccc} C_{\left\{x_{i};-T\right\},\left\{X^{j}\right\}} & = & {\left[\frac{dQ}{dT}\right]}_{\left\{x_{i};T\right\},\left\{X^{j}\right\}}={\left[\frac{\partial U}{\partial x_{\alpha }}\right]}_{\left\{x_{i};T\right\},\left\{X^{j}\right\}}-x_{\lambda }{\left[\frac{\partial X^{\lambda }}{\partial x_{\alpha }}\right]}_{\left\{x_{i};-T\right\}\left\{X^{j}\right\}}\\ & = & a_{0}^{i}\;\mathrm{for}\;i\leq n. \end{array}$$

Therefore, $a_{0}^{3}=C_{\mu ,V}$ and $a_{0}^{4}=C_{\mu ,N}$. In this case, it is possible from the 3- and 4-equations to get the 7-equation, which could previously be calculated using Equations (15) and (16). By putting $a_{0}^{7}={\left(\frac{\partial U}{\partial T}\right)}_{V,N}=C_{V,N}$ in the 7-equation, we obtain

(28) $$TdS=C_{V,N}dT+\frac{NkT}{V}dV+\left[\frac{3}{2}kT-\mu \right]dN.$$

We have

$$a_{0}^{7}=\frac{a_{2}^{4}a_{0}^{3}-a_{2}^{3}a_{0}^{4}}{a_{2}^{4}-a_{2}^{3}},\;A_{1}^{7}=\frac{A_{1}^{3}a_{2}^{4}}{a_{2}^{4}-a_{2}^{3}},\;\mathrm{and}\;A_{2}^{7}=-\frac{A_{2}^{4}a_{2}^{3}}{a_{2}^{4}-a_{2}^{3}}.$$

Then, by substituting the above calculated coefficients, we arrive at

$$a_{0}^{7}=\frac{-C_{\mu ,V}-\left[\frac{3}{2}-\frac{\mu }{kT}\right]C_{\mu ,N}}{\left[\frac{\mu }{kT}-\frac{5}{2}\right]},\;A_{1}^{7}=\frac{NkT}{V},\;\mathrm{and}\;A_{2}^{7}=\left[\frac{3}{2}kT-\mu \right].$$

We obtain

(29) $$\left[\frac{3}{2}-\frac{\mu }{kT}\right]\frac{C_{\mu ,N}}{C_{\mu ,V}}+1=-\frac{C_{V,N}}{C_{\mu ,V}}\left[\frac{\mu }{kT}-\frac{5}{2}\right],$$

which represents a novel identity.

The 9-equation is (see Appendix B):

(30) $$\begin{array}{ccc} TdS & = & \left[-\frac{N}{W(Z)}+\frac{N}{\left(1+W(Z)\right)W\left(Z\right)}\right]d\mu +\left[\frac{2\mu N}{3V}\left(-\frac{1}{W(Z)}+\frac{1}{W\left(Z\right)+W^{2}\left(Z\right)}\right)\right]dV\\ & & +\left[\frac{1}{3}\mu \left(-3-\frac{3}{W(Z)}-\frac{2}{W\left(Z\right)+W^{2}\left(Z\right)}\right)\right]dN. \end{array}$$

Now we can recalculate the 9-equation using the 3- and 7-equations. We arrive at

$$a_{2}^{9}=\frac{a_{0}^{7}a_{2}^{3}}{\left(a_{0}^{7}-a_{0}^{3}\right)},\;A_{1}^{9}=\frac{\left(A_{1}^{3}a_{0}^{7}-A_{1}^{7}a_{0}^{3}\right)}{\left(a_{0}^{7}-a_{0}^{3}\right)},\;\mathrm{and}\;A_{2}^{9}=-\frac{a_{0}^{3}A_{2}^{7}}{\left(a_{0}^{7}-a_{0}^{3}\right)}.$$

That is,

$$\begin{array}{ccc} N\left(-\frac{1}{W(Z)}+\frac{1}{\left(1+W(Z)\right)W\left(Z\right)}\right) & = & \frac{C_{V,N}\left[\frac{3}{2}N-\frac{\mu N}{kT}\right]}{C_{V,N}-C_{\mu ,V}},\\ \frac{2\mu N}{3V}\left(-\frac{1}{W(Z)}+\frac{1}{W\left(Z\right)+W^{2}\left(Z\right)}\right) & = & \frac{NkT}{V}\left\{\frac{\left[\frac{5}{2}-\frac{\mu }{kT}\right]}{1-\frac{C_{\mu ,V}}{C_{V,N}}}-\frac{1}{\frac{C_{V,N}}{C_{\mu ,V}}-1}\right\},\\ \frac{1}{3}\mu \left(-3-\frac{3}{W(Z)}-\frac{2}{W\left(Z\right)+W^{2}\left(Z\right)}\right) & = & \frac{C_{\mu ,V}\left[\mu -\frac{3}{2}kT\right]}{C_{V,N}-C_{\mu ,V}}. \end{array}$$

Obtaining from the last identity

(31) $$C_{V,N}=-\frac{3kT}{\mu }\frac{\left(-\frac{1}{W(Z)}+\frac{1}{\left(1+W(Z)\right)W\left(Z\right)}\right)}{\left(-3-\frac{3}{W(Z)}-\frac{2}{W\left(Z\right)+W^{2}\left(Z\right)}\right)}C_{\mu ,V}.$$

This is a novel identity, not reported in the literature, and it represents an interesting result since normally, the heat capacity at constant μ and *V* is never taken into account.

Another example is using the 8-equation, the 2-equation, and the 7-equation, obtaining

$$C_{P,N}-C_{V,N}=\frac{2}{3}C_{V,N},$$

which corresponds to the Mayer relation when *N* is constant.

### 4.2. Unconventional System: A Particular Case

As we mentioned in the Introduction, it has been shown that for thermodynamic systems with unconventional state equations, the Carnot theorem is valid [6]. Apparently, the Carnot theorem is a universal property for a large number of systems despite not behaving physically.

Let us consider the following equation of state,

(32) $$P=\frac{aV}{NT},$$

with a>0. We have to note that the isothermal compressibility $\kappa_{T}$,

(33) $$\kappa_{T}=-\frac{1}{V}{\left(\frac{\partial V}{\partial P}\right)}_{T,N}=-\frac{1}{V}\frac{NT}{a}=-\frac{NT}{\frac{PNT}{a}a}=-\frac{1}{P}<0,$$

is negative and for most substances $\kappa_{T}>0$. In statistical mechanics, it is required that $\kappa_{T}>0$ in order to have low fluctuations which give sense to it. Moreover, Van Hove’s theorem [1] proves that by means of regular intermolecular forces, $\kappa_{T}$ must be positive within the framework of statistical mechanics. However, as we shall see, we can have a thermodynamic system without matching the classical proposal of statistical mechanics. A discussion about this point is conducted at the end of the Hessian method.

On the other hand, as we do not have another equation of state, we use our method for proposing it. First, we know that

$${\left(\frac{\partial U}{\partial V}\right)}_{T}=T{\left(\frac{\partial P}{\partial T}\right)}_{V}-P.$$

In our case,

$${\left(\frac{\partial U}{\partial V}\right)}_{T,N}=T\left(-\frac{aV}{NT^{2}}\right)-\frac{aV}{NT}=-\frac{2aV}{NT}.$$

We know that

$${\left(\frac{\partial^{2}U}{\partial V\partial T}\right)}_{N}={\left(\frac{\partial^{2}U}{\partial T\partial V}\right)}_{N},$$

Then, we can propose that *U* is such that

(34) $$U=-\frac{aV^{2}}{NT}\;\mathrm{or}\;U=-PV,$$

which shows that the internal energy is always negative. Note that in order to differentiate our system with respect to regular systems, we do not incorporate the ideal gas limit term $\frac{3}{2}NRT$ in order to have a completely different unconventional system.

#### 4.2.1. Calculation of Entropy with the *N* Variable

From Equation (32), by integrating the Gibbs–Duhem relation, we can calculate the chemical potential obtaining

(35) $$\frac{\mu }{T}=\frac{u^{2}}{2av^{2}}-\frac{u_{0}^{2}}{2av_{0}^{2}}+{\left(\frac{\mu }{T}\right)}_{0},$$

where u=U/N and v=V/N. On the other hand, by applying the Euler relation, we arrive at

$$s=\frac{1}{T}u+\frac{P}{T}v-\frac{\mu }{T},$$

Therefore, the entropy is

(36) $$S=-\frac{NU^{2}}{2aV^{2}}+Ns_{0},$$

where

$$s_{0}=\frac{u_{0}^{2}}{2av_{0}^{2}}-{\left(\frac{\mu }{T}\right)}_{0}.$$

We obtain an entropy that complies with the first-degree homogeneous property. However, it has to be noted that entropy can be negative. This implies that we obtain a restriction in the value of the chemical potential μ of this system. Indeed, It is important to note that if we rewrite *S*, as S(T,μ,N), using Equation (35) in the following form, we have

(37) $$\begin{array}{ccc} S & = & -\frac{NU^{2}}{2aV^{2}}+N\left(\frac{u_{0}^{2}}{2av_{0}^{2}}-{\left(\frac{\mu }{T}\right)}_{0}\right)\\ & = & -\frac{N\mu }{T}. \end{array}$$

Therefore, in order to have a positive entropy S>0, we need to have a negative chemical potential, μ<0.

#### 4.2.2. Hessian Method

We can obtain the internal energy as a fundamental equation using Equation (36)

(38) $$U=-\frac{\sqrt{2a}V\sqrt{Ns_{0}-S}}{\sqrt{N}}.$$

Since from Equation (36),

$$Ns_{0}-S=\frac{NU^{2}}{2aV^{2}}>0,$$

we can assure that the internal energy is a real negative quantity. First, note that the temperature

(39) $${\left[\frac{\partial U}{\partial S}\right]}_{V,N}=\frac{\sqrt{a}V}{\sqrt{2}\sqrt{N}\sqrt{Ns_{0}-S}}=T>0.$$

We can calculate the Hessian by noting that Shwarz’s theorem is fulfilled. Then, from the the Hessian, we obtain the characteristic polynomial,

$$\begin{array}{c} \begin{array}{c} \left(\begin{array}{c} 4N^{4}S^{2}+4N^{2}S^{4}-8N^{5}Ss_{0}-8N^{3}S^{3}s_{0}+4N^{6}s_{0}^{2}\\ +4N^{4}S^{2}s_{0}^{2}+4N^{2}S^{2}V^{2}-8N^{3}Ss_{0}V^{2}+4N^{4}s_{0}^{2}V^{2} \end{array}\right)\lambda \\ +\left(N^{2}V-3S^{2}V+4NSs_{0}V\right)\lambda^{2}\\ -\lambda^{3}=0 \end{array} \end{array}$$

We can see that one of the eigenvalues is λ=0, and the other two are,

$$\lambda_{\pm }=\alpha \pm \beta ,$$

with

$$\alpha =\frac{1}{2}\left(N^{2}V+\left(4Ns_{0}-3S\right)SV\right)$$

and

$$\beta =\frac{1}{2}{\left[{\left(N^{2}V-3S^{2}V+4NSs_{0}V\right)}^{2}+16N^{2}\left(\begin{array}{c} N^{2}S^{2}+S^{4}-2N^{3}Ss_{0}-2NS^{3}s_{0}+N^{4}s_{0}^{2}\\ +N^{2}S^{2}s_{0}^{2}+S^{2}V^{2}-2NSs_{0}V^{2}+N^{2}s_{0}^{2}V^{2} \end{array}\right)\right]}^{\frac{1}{2}}.$$

We know that $Ns_{0}>S$, and it must be fulfilled that S>0, so for $Ns_{0}>0$, we can see

$$4Ns_{0}-3S>0,$$

so α>0. Rewriting β, we have

$$\beta =\frac{1}{2}{\left[{\left(N^{2}V-3S^{2}V+4NSs_{0}V\right)}^{2}+16N^{2}\left({\left(NS-N^{2}s_{0}\right)}^{2}+{\left(S^{2}-NSs_{0}\right)}^{2}+{\left(SV-Ns_{0}V\right)}^{2}\right)\right]}^{\frac{1}{2}},$$

so β>0.

However, this system will be an unstable system according to the Le Chatelier–Braun principle [4] because the isothermal compressibility is negative (see Equation (33)). However, the no-null eigenvalues are real, which means that the relaxation times τ∝1/λ [9] are real. This means that this system is an unstable thermodynamic system that may after a perturbation come back to a equilibrium state [10], but the effects of the instability will bring it to a position of increasing volume.

#### 4.2.3. Entropy Method

By looking at Equations (32), (34), (36) and (39), we have already proven that the items **A**, **F**, **G**, **H**, and **I** are fulfilled. Therefore, the set of equations represents a thermodynamic system.

#### 4.2.4. Completeness Method

In general, we can express the TdS equations of this system using Equation (15). In this case, we need just three independent TdS equations. We choose the 1-, 2-, and 3-equations.

The 1-equation is:

(40) $$TdS=a_{0}^{1}dT+a_{1}^{1}dP+A_{1}^{1}dV,$$

From Equations (15) and (16), we need to know $U=U\left(T,P,V\right)$ and $N=N\left(T,P,V\right)$, that is:

$$N=\frac{aV}{PT},\;U=-VP\;\mathrm{and}\;\mu =\frac{TP^{2}}{2a}+CT,$$

with $C=-\frac{u_{0}^{2}}{2av_{0}^{2}}+{\left(\frac{\mu }{T}\right)}_{0}$. We arrive to the 1-equation,

(41) $$TdS=C_{P,V}dT+\left(-\frac{V}{2}+\frac{CaV}{P^{2}}\right)dP+\left(-\frac{P}{2}-\frac{Ca}{P}\right)dV,$$

with $C_{P,V}=\frac{PV}{2T}+\frac{CaV}{PT}$.

The 2-equation is:

(42) $$TdS=a_{0}^{2}dT+a_{1}^{2}dP+A_{2}^{2}dN.$$

From Equations (15) and (16), we need to know $U=U\left(T,P,N\right)$ y $V=V\left(T,P,N\right)$, that is:

$$V=\frac{TPN}{a},\;U=-\frac{TP^{2}N}{a}\;\mathrm{and}\;\mu =\frac{TP^{2}}{2a}+CT.$$

Finally, the 2-equation is

(43) $$TdS=-\frac{TPN}{a}dP+\left(-\frac{TP^{2}}{2a}-CT\right)dN.$$

The 3-equation is:

(44) $$TdS=a_{0}^{3}dT+a_{2}^{3}d\mu +A_{1}^{3}dV,$$

From Equations (15) and (16), we need to know $U=U\left(T,\mu ,V\right)$, $N=N\left(T,\mu ,V\right)$, and $P=P\left(T,\mu ,V\right)$, that is:

$$N=\frac{aV}{\sqrt{2}T^{\frac{1}{2}}{\left(a\mu -aCT\right)}^{\frac{1}{2}}},\;U=-\frac{V\sqrt{2}{\left(a\mu -aCT\right)}^{\frac{1}{2}}}{T^{\frac{1}{2}}}\;\mathrm{and}\;P=\frac{\sqrt{2}{\left(a\mu -aCT\right)}^{\frac{1}{2}}}{T^{\frac{1}{2}}}.$$

We need to analyze μ−CT; we know that $\mu =\frac{u^{2}T}{2av^{2}}-\frac{u_{0}^{2}T}{2av_{0}^{2}}+T{\left(\frac{\mu }{T}\right)}_{0}$ and $C=-\frac{u_{0}^{2}}{2av_{0}^{2}}+{\left(\frac{\mu }{T}\right)}_{0}$, we see that $\mu -CT=\frac{u^{2}T}{2av^{2}}$, and μ−CT>0, so ${\left(a\mu -aCT\right)}^{\frac{1}{2}}$ is real. Therefore, the 3-equation is

(45) $$TdS=C_{\mu ,V}dT+\frac{-a^{2}\mu V+2a^{2}VCT}{2\sqrt{2}T^{\frac{1}{2}}{\left(a\mu -aCT\right)}^{\frac{3}{2}}}d\mu -\frac{a\mu }{\sqrt{2}T^{\frac{1}{2}}{\left(a\mu -aCT\right)}^{\frac{1}{2}}}dV,$$

with $a_{0}^{3}=C_{\mu ,V}=\frac{a\mu V}{2\sqrt{2}T^{\frac{3}{2}}{\left(a\mu -aCT\right)}^{\frac{1}{2}}}\left(\frac{3a\mu -4aCT}{a\mu -aCT}\right)$.

##### Thermodynamic Identities

With the three independent TdS equations, it is possible to calculate the remaining TdS equations and in this way obtain different thermodynamic identities. In this case, it is possible from the 1-equation and 2-equation to deduce the 7-equation, which can be calculated previously with the formulas of the method developed in Section 3. We have

$$a_{0}^{7}=\frac{a_{1}^{2}a_{0}^{1}-a_{1}^{1}a_{0}^{2}}{a_{1}^{2}-a_{1}^{1}},\;A_{1}^{7}=\frac{A_{1}^{1}a_{1}^{2}}{a_{1}^{2}-a_{1}^{1}},\;\mathrm{and}\;A_{2}^{7}=-\frac{A_{2}^{2}a_{1}^{1}}{a_{1}^{2}-a_{1}^{1}}.$$

Then, if we substitute the already calculated coefficients in $a_{0}^{7}={\left(\frac{\partial U}{\partial T}\right)}_{V,N}=C_{V,N}$ (remember that $a_{0}^{2}=C_{P,N}=0$), we have

(46) $$\left(C_{V,N}-C_{P,V}\right)=C_{V,N}\left(\frac{aVP^{2}-2a^{2}VC}{2TP^{3}N}\right).$$

Note that it can be proven that this system complies with the Carnot theorem.

### 4.3. The Rubber Band

The rubber band is normally described using two equations of motion which consider the following constraints:

(*i*) An analogue of the mole number might be associated with the number of monomer units in the rubber band, but in this first approach, this mole number is not considered as a variable and is taken as a constant. It has to be highlighted that for the ideal gas, we can consider the number of particles as a constant, but the entropy will not be a homogeneous function of degree one. The same happens with the entropy of the rubber band when the number of monomer is not taken as a variable.
(*ii*) In the rubber band, the length *L* and the tension τ play a role analogue to the volume *V* and minus the pressure −P in the ideal gas, respectively.
(*iii*) $L_{0}$ represents the unstretched length of the rubber band, $L_{1}$ is the elastic limit length, and *c* and *b* are characteristic constants.

The equations of state for this model are:

(47) $$\tau =bT\frac{L-L_{0}}{L_{1}-L_{0}},$$

and

(48) $$U=cL_{0}T.$$

By considering the different representations from the variables *T*, τ, and *L*, we can obtain the TdS equations, to check the validity of the entropic relations and deduce the entropy. Everything functions like a thermodynamic system unless the entropy does not satisfy the first-degree homogeneous property; that is, the obtained entropy is

(49) $$S=S_{0}+cL_{0}\ln\frac{U}{U_{0}}-b\frac{L-L_{0}}{L_{1}-L_{0}}dL.$$

which is obviously not a first-degree homogeneous function as happens with the ideal gas when the number of particles *N* is taken as a constant. It has to be noted that Callen [4] says that these equations have been constructed on the basis only of the most qualitative of information. They do not include any numbers of monomers. Our purpose in this section is to give an expression of the entropy which meets the first-degree homogeneous requirement. However, as in the ideal gas, we are obligated to consider including a quantity related with the number of monomer. Let us consider that this number of monomer can be described by *N* in a linear way, $L_{0}/N_{0}$ being the length of each monomer when the rubber band measures $L_{0}$ and L/N the length of each monomer when the rubber band measures *L* and is composed of *N* monomers.

Therefore, let us propose the following equations of state with *N* variable:

(50) $$\tau =\frac{b}{a}T\left(\frac{L}{N}-\frac{L_{0}}{N_{0}}\right),$$

with

$$a=L_{1}-L_{0}$$

Then, we need to propose another equation of state. We propose it using the property that *U* is a function of state and therefore,

$$dU={\left(\frac{\partial U}{\partial L}\right)}_{T}dL+{\left(\frac{\partial U}{\partial T}\right)}_{L}dT.$$

and

(51) $${\left(\frac{\partial^{2}U}{\partial L\partial T}\right)}_{N}={\left(\frac{\partial^{2}U}{\partial T\partial L}\right)}_{N},$$

We arrive at

(52) $$\frac{\partial^{2}U}{\partial T\partial L}=0.$$

We can now propose

$$C_{V,N}={\left(\frac{\partial U}{\partial T}\right)}_{V,N}=c\frac{N}{N_{0}}L_{0}.$$

Consequently, we arrive to our final proposal for the internal energy as

(53) $$U=cNl_{0}T,$$

where $l_{0}=L_{0}/N_{0}$, and it coincides with the internal energy when the number of monomers are constant equal to $N_{0}$.

#### 4.3.1. Calculation of Entropy with N Variable

The state equations for this case are Equations (50) and (53)

$$\tau =\frac{b}{a}T\left(\frac{L}{N}-l_{0}\right)\;\mathrm{and}\;U=cNl_{0}T.$$

From these expressions, using the Gibbs–Duhem method, we obtain the entropy,

(54) $$S=\frac{b}{2a}\frac{L^{2}}{N}-\frac{b}{a}l_{0}L+Ncl_{0}\ln\left(\frac{U}{N}\right)+Ns_{0},$$

where,

$$s_{0}=cl_{0}-cl_{0}\ln u_{0}+\frac{b}{2a}l_{0}^{2}+{\left(\frac{\mu }{T}\right)}_{0}.$$

The expression for the entropy shows that is a first-degree homogeneous function. Moreover, $\mu_{0}$ must be such that S>0. Therefore, this will limit the validity of the system by giving a range of temperature such that S>0.

We can explicitly write the energy in the following way

(55) $$U=N\exp\left\{\frac{S-Ns_{0}}{Ncl_{0}}-\frac{b}{2acl_{0}}\frac{L^{2}}{N^{2}}+\frac{b}{ac}\frac{L}{N}\right\}.$$

#### 4.3.2. Hessian Method

We rewrite our energy as

(56) $$U=N\exp\left\{A\frac{S}{N}-B-X\frac{L^{2}}{N^{2}}+Y\frac{L}{N}\right\},$$

with $A=\frac{1}{cl_{0}}$, $B=\frac{s_{0}}{cl_{0}}$, $X=\frac{b}{2acl_{0}}$ y $Y=\frac{b}{ac}$.

First, it is necessary to calculate the temperature,

(57) $${\left[\frac{\partial U}{\partial S}\right]}_{L,N}=\frac{A}{N}N\exp\left\{A\frac{S}{N}-B-X\frac{L^{2}}{N^{2}}+Y\frac{L}{N}\right\}=\frac{A}{N}U=T.$$

This shows the requirement that ${\left[\frac{\partial U}{\partial S}\right]}_{L,N}=T>0$.

Further, it is clear that the internal energy accomplishes Schwarz’s theorem.

Then, the characteristic polynomial of the Hessian is

$$\left(\begin{array}{c} 2A^{2}L^{2}N^{6}X+2A^{2}N^{8}X\\ +2A^{2}N^{6}S^{2}X \end{array}\right)\lambda +\left(\begin{array}{c} A^{2}N^{4}+A^{2}N^{2}S^{2}\\ -2L^{2}N^{2}X-2N^{4}X\\ -4AL^{2}NSX+4L^{4}X^{2}\\ +4L^{2}N^{2}X^{2}+2ALN^{2}SY\\ -4L^{3}NXY-4LN^{3}XY\\ +L^{2}N^{2}Y^{2}+N^{4}Y^{2} \end{array}\right)\lambda^{2}-\lambda^{3}=0.$$

We can see that one of the eigenvalues is λ=0 and the other two are

$$\lambda_{\pm }=\alpha \pm \beta ,$$

with

$$\alpha =\frac{1}{2}\left(\begin{array}{c} A^{2}N^{4}+A^{2}N^{2}S^{2}-2L^{2}N^{2}X-2N^{4}X\\ -4AL^{2}NSX+4L^{4}X^{2}+4L^{2}N^{2}X^{2}+2ALN^{2}SY\\ -4L^{3}NXY-4LN^{3}XY+L^{2}N^{2}Y^{2}+N^{4}Y^{2} \end{array}\right)$$

and

$$\beta =\frac{1}{2}{\left[\begin{array}{c} {\left(\begin{array}{c} A^{2}N^{4}+A^{2}N^{2}S^{2}-2L^{2}N^{2}X-2N^{4}X\\ -4AL^{2}NSX+4L^{4}X^{2}+4L^{2}N^{2}X^{2}+2ALN^{2}SY\\ -4L^{3}NXY-4LN^{3}XY+L^{2}N^{2}Y^{2}+N^{4}Y^{2} \end{array}\right)}^{2}\\ +\left(8A^{2}L^{2}N^{6}X+8A^{2}N^{8}X+8A^{2}N^{6}S^{2}X\right) \end{array}\right]}^{\frac{1}{2}}.$$

Consequently, the requirement for the Hessian is satisfied.

#### 4.3.3. Entropy Method

By looking at Equations (50), (53), (54) and (57), we have already proven that the items **A**, **G**, **C**, **H**m and **I** are fulfilled. Therefore, the set of equations represents a thermodynamic system.

#### 4.3.4. Completeness Method

In general, we can express all the TdS equations of this system using Equation (15). Before continuing, let us give the chemical potential as:

$$\begin{array}{ccc} \mu & = & -cTl_{0}\ln\frac{U}{N}-\frac{bT}{2a}\frac{L^{2}}{N^{2}}+cl_{0}T-Ts_{0},\\ \mu & = & -cTl_{0}\ln\frac{U}{N}-\frac{bT}{2a}\frac{L^{2}}{N^{2}}+TC \end{array}$$

with

$$C=cl_{0}\ln u_{0}+\frac{b}{2a}l_{0}^{2}+{\left(\frac{\mu }{T}\right)}_{0}.$$

As in the case of the ideal gas from the ten TdS equations, we just need to choose three linearly independent equations. Let us choose the 1-equation, the 2-equation, and the 3-equation.

The 1-equation is:

$$TdS=a_{0}^{1}dT+a_{1}^{1}d\tau +A_{1}^{1}dL.$$

From Equations (15) and (16), we need $U=U\left(T,\tau ,L\right)$ and $N=N\left(T,\tau ,L\right)$. We have

$$U=cl_{0}TL{\left(\frac{a\tau }{bT}+l_{0}\right)}^{-1}\;\mathrm{and}\;N=L{\left(\frac{a\tau }{bT}+l_{0}\right)}^{-1},$$

and

$$\mu =-cl_{0}T\ln cl_{0}T-\frac{bT}{2a}{\left(\frac{a\tau }{bT}+l_{0}\right)}^{2}+TC.$$

Therefore,

(58) $$\begin{array}{ccc} a_{0}^{1} & = & C_{\tau ,L}=\left[1+\ln cl_{0}T-\frac{C}{cl_{0}}\right]\frac{acLl_{0}\tau }{bT}{\left(\frac{a\tau }{bT}+l_{0}\right)}^{-2}+cLL_{0}{\left(\frac{a\tau }{bT}+l_{0}\right)}^{-1}+\frac{L\tau }{2T},\\ a_{1}^{1} & = & -\left[1+\ln cl_{0}T-\frac{C}{cl_{0}}\right]\frac{acLl_{0}}{b}{\left(\frac{a\tau }{bT}+l_{0}\right)}^{-2}-\frac{L}{2},\\ A_{1}^{1} & = & \left[1+\ln cl_{0}T-\frac{C}{cl_{0}}\right]cl_{0}T{\left(\frac{a\tau }{bT}+l_{0}\right)}^{-1}-\frac{\tau }{2}+\frac{bT}{2a}l_{0}. \end{array}$$

The 2-equation is:

$$TdS=a_{0}^{2}dT+a_{1}^{2}d\tau +A_{2}^{2}dN,$$

From Equations (15) and (16), we need to know $U=U\left(T,\tau ,N\right)$ and $L=L\left(T,\tau ,N\right)$. We have

$$U=cl_{0}TN\;\mathrm{and}\;L=N\left(\frac{a\tau }{bT}+l_{0}\right),$$

and

$$\mu =-cl_{0}T\ln cl_{0}T-\frac{bT}{2a}{\left(\frac{a\tau }{bT}+l_{0}\right)}^{2}+TC.$$

Therefore,

(59) $$\begin{array}{ccc} a_{0}^{2} & = & cl_{0}N+\frac{aN\tau^{2}}{bT^{2}},\;a_{1}^{2}=-\frac{aN\tau }{bT},\\ A_{2}^{2} & = & cl_{0}T\left(1+\ln cl_{0}T-\frac{C}{cl_{0}}\right)-\tau \left(\frac{a\tau }{bT}+l_{0}\right)+\frac{bT}{2a}{\left(\frac{a\tau }{bT}+l_{0}\right)}^{2}. \end{array}$$

Hence,

(60) $$TdS=C_{\tau ,N}dT-\frac{aN\tau }{bT}d\tau +A_{2}^{2}dN,$$

with $a_{0}^{2}=C_{\tau ,N}=cl_{0}N+\frac{aN\tau^{2}}{bT^{2}}$.

The 3-equation is:

(61) $$TdS=a_{0}^{3}dT+a_{2}^{3}d\mu +A_{1}^{3}dL,$$

From Equations (15) and (16), we need to know $U=U\left(T,\mu ,L\right)$, $N=N\left(T,\mu ,L\right)$ and $\tau =\tau \left(T,\mu ,L\right)$. We have

$$N=\frac{b^{\frac{1}{2}}LT^{\frac{1}{2}}}{\sqrt{2}{\left(-a\mu +aCT-acl_{0}T\ln cl_{0}T\right)}^{\frac{1}{2}}},\;U=\frac{cl_{0}b^{\frac{1}{2}}LT^{\frac{3}{2}}}{\sqrt{2}{\left(-a\mu +aCT-acl_{0}T\ln cl_{0}T\right)}^{\frac{1}{2}}},$$

and

$$\tau =\frac{bT}{a}\left(\frac{\sqrt{2}{\left(-a\mu +aCT-acl_{0}T\ln cl_{0}T\right)}^{\frac{1}{2}}}{b^{\frac{1}{2}}T^{\frac{1}{2}}}-l_{0}\right).$$

Accordingly,

(62) $$\begin{array}{c} \begin{array}{ccc} a_{0}^{3}=C_{\mu ,L} & = & -\frac{b^{\frac{1}{2}}cLl_{0}T^{\frac{3}{2}}\left(aC-acl_{0}-acl_{0}\ln cl_{0}T\right)}{2\sqrt{2}{\left(-a\mu +aCT-acl_{0}T\ln cl_{0}T\right)}^{\frac{3}{2}}}\left(1-\frac{\mu }{cl_{0}T}\right)\\ & & +\frac{3b^{\frac{1}{2}}cLl_{0}T^{\frac{1}{2}}}{2\sqrt{2}{\left(-a\mu +aCT-acl_{0}T\ln cl_{0}T\right)}^{\frac{1}{2}}}\left(1-\frac{\mu }{3cl_{0}T}\right),\\ a_{2}^{3} & = & \frac{ab^{\frac{1}{2}}cLl_{0}T^{\frac{3}{2}}}{2\sqrt{2}{\left(-a\mu +aCT-acl_{0}T\ln cl_{0}T\right)}^{\frac{3}{2}}}\left(1-\frac{\mu }{cl_{0}T}\right),\\ A_{1}^{3} & = & \frac{b^{\frac{1}{2}}cl_{0}T^{\frac{3}{2}}}{\sqrt{2}{\left(-a\mu +aCT-acl_{0}T\ln cl_{0}T\right)}^{\frac{3}{2}}}\left(1-\frac{\mu }{cl_{0}T}\right)\\ & & -\frac{bT}{a}\left(\frac{\sqrt{2}{\left(-a\mu +aCT-acl_{0}T\ln cl_{0}T\right)}^{\frac{1}{2}}}{b^{\frac{1}{2}}T^{\frac{1}{2}}}-l_{0}\right), \end{array} \end{array}$$

We know that with the three above TdS equations, we can generate all the other TdS equations. We deal with the 7-equation in order to determine some interesting identities. The 7-equation is:

(63) $$TdS=a_{0}^{7}dT+A_{1}^{7}dL+A_{2}^{7}dN,$$

with

$$a_{0}^{7}={\left(\frac{\partial U}{\partial T}\right)}_{L,N},\;A_{1}^{7}={\left(\frac{\partial U}{\partial L}\right)}_{T,N}-\tau \;\mathrm{and}\;A_{2}^{7}={\left(\frac{\partial U}{\partial N}\right)}_{T,L}-\mu .$$

From Equations (15) and (16), we need to know $U=U\left(T,L,N\right)$, $\tau =\tau \left(T,L,N\right)$ and $\mu =\mu \left(T,L,N\right)$. We have

$$U=cNl_{0}T,\;\tau =\frac{b}{a}T\left(\frac{L}{N}-l_{0}\right),\;\mathrm{and}\;\mu =-cTl_{0}\ln cl_{0}T-\frac{bT}{2a}\frac{L^{2}}{N^{2}}+TC.$$

Therefore,

(64) $$a_{0}^{7}=cl_{0}N,\;A_{1}^{7}=-\frac{b}{a}T\left(\frac{L}{N}-l_{0}\right),\;A_{2}^{7}=cl_{0}T+cTl_{0}\ln cl_{0}T+\frac{bT}{2a}\frac{L^{2}}{N^{2}}-TC.$$

with $a_{0}^{7}=C_{L,N}=cl_{0}N.$

##### Thermodynamic Identities

With the three linearly independent TdS equations, it is possible to calculate the remaining TdS equations and in this way obtain different thermodynamic identities. In this case, it is possible from the 1-equation, Equation (58), and the 2-equation, Equation (60), to deduce the 7-equation, Equations (63) and (64), using the method previously described in Section 3; we obtain

(65) $$a_{0}^{7}=\frac{a_{1}^{2}a_{0}^{1}-a_{1}^{1}a_{0}^{2}}{a_{1}^{2}-a_{1}^{1}},\;A_{1}^{7}=\frac{A_{1}^{1}a_{1}^{2}}{a_{1}^{2}-a_{1}^{1}},\;A_{2}^{7}=-\frac{A_{2}^{2}a_{1}^{1}}{a_{1}^{2}-a_{1}^{1}}.$$

Then, we arrive at

(66) $$C_{L,N}=a_{0}^{7}=\frac{a_{1}^{2}C_{\tau ,L}-a_{1}^{1}C_{\tau ,N}}{a_{1}^{2}-a_{1}^{1}},\;A_{1}^{7}=\frac{A_{1}^{1}a_{1}^{2}}{a_{1}^{2}-a_{1}^{1}},\;A_{2}^{7}=-\frac{A_{2}^{2}a_{1}^{1}}{a_{1}^{2}-a_{1}^{1}}.$$

Finally, a Mayer-like equation for the rubber band is obtained:

(67) $$C_{\tau ,L}-C_{L,N}=\left(\left[1+\ln cl_{0}T-\frac{C}{cl_{0}}\right]\frac{cLl_{0}T}{N\tau }{\left(\frac{a\tau }{bT}+l_{0}\right)}^{-2}+\frac{bTL}{2aN\tau }\right)\left(C_{\tau ,N}-C_{L,N}\right).$$

### 4.4. The Paramagnetic Solid

There exists an interesting discussion about the TdS equations for a paramagnetic solid [11]. Indeed, according to Kittel [12] “a great deal of unnecessary confusion exists as to how to write the First Law of Thermodynamics for a magnetic system”. Callen [4] gave a proposal for a simple paramagnetic system described by the internal energy as:

$$U=NkT_{0}\left[\frac{S}{Nk}+\frac{M^{2}}{N^{2}M_{0}^{2}}\right],$$

where *M* represents the magnetization, and $T_{0}$ and $M_{0}$ are positive constants. However, he said that this model does not describe any particular known system. Moreover, in Appendix C, he treated the paramagnetic solid by including the solenoid which produces the magnetic field, redefining the energy of the system by putting

$$U=E-\frac{1}{2\mu_{0}}\int B^{2}dV.$$

However, he needed to give a redefinition of the energy by subtracting the energy stored in the volume by the magnetic field. The most appropriate treatment is given by Barrett and Macdonald [13], who studied the system in a more direct way and through statistical physics obtain an expression for the work done by the magnetic field in a paramagnetic solid. Barrett and Macdonald mentioned there are two forms for the work done when the magnetic field *B* and the magnetization *M* change, that is:

(68) $$\delta_{ms}W=BdM\;and\;\delta_{s}W=-MdB,$$

where the form $\delta_{ms}W$ applies when the mutual field energy is included in the system and the form $\delta_{s}W$ when it is not. Although he claimed that there are thermodynamic systems with no fundamental thermodynamic equation, he arrived to the conclusion that the work done by the magnetic field for a system where the mutual field is not included as

(69) $$\delta_{s}W=-MdB.$$

Of course, for a fundamental equation in the *S* representation, the work must be written as the product of an intensive quantity times the differential of an extensive quantity, which is not expressed in Equation (69) (note that there is a misprint in the Barrett and Macdonald [13] article of the sign in the expression for the temperature). The good expression is:

(70) $$\begin{array}{ccc} \frac{\mu B}{kT} & = & \ln\left[\mu +M/N\right]-\ln\left[\mu -M/N\right]. \end{array}$$

(71) $$\begin{array}{cc} = & \ln\left[\frac{1+M/\mu N}{1-M/\mu N}\right] \end{array}$$

(72) $$\begin{array}{cc} = & \tanh^{-1}M/\mu N. \end{array}$$

From Equation (70), he arrived to the correct expression for the entropy:

(73) $$S=Nk\left[-\left(\frac{1}{2}+\frac{M}{2\mu N}\right)\ln\left(\frac{1}{2}+\frac{M}{2\mu N}\right)-\left(\frac{1}{2}-\frac{M}{2\mu N}\right)\ln\left(\frac{1}{2}-\frac{M}{2\mu N}\right)\right],$$

or in a different form

(74) $$S=-\frac{Nk}{2}\left[\left(1+\frac{M}{\mu N}\right)\ln\left(1+\frac{M}{\mu N}\right)+\left(1-\frac{M}{\mu N}\right)\ln\left(1-\frac{M}{N}\right)\right]+Nk\ln2$$

being S=S(M,N) and consequently

(75) $${\left[\frac{\partial S}{\partial U}\right]}_{M,N}=\frac{1}{T}=0,$$

which implies an infinite temperature. These contradicts Equation (70), where the temperature is well-defined. Therefore, we need to obtain another representation to express the thermodynamic system. The problem is based on that the TdS equation is written as

(76) TdS=dU+MdB,

which implies

(77) $${\left[\frac{\partial S}{\partial N}\right]}_{U,B}=\lambda =0\;\mathrm{and}\;{\left[\frac{\partial S}{\partial B}\right]}_{U,N}=\frac{M}{T},$$

where $\lambda ={\left(\partial S/\partial N\right)}_{U,B}$ does not represent minus the chemical potential over the temperature $-\phi /T={\left(\partial S/N\right)}_{U,X}$ (we use this notation for this case in order to not be confused with the magnetic μ, and *X* represents an extensive quantity which has to be defined below). That is, these identities derived from Equation (76) are obtained by considering that the number of particles is constant and can be corrected by calculating the chemical potential using the Gibbs–Duhem relation. However, due to Equations (73)–(75), we can conclude that the magnetization *M* is not a good extensive variable.

In order to solve this inconsistency, let us define a set of extensive variables for the paramagnetic solid: *U* the internal energy, *N* the number of particles and

**Figure 1:**
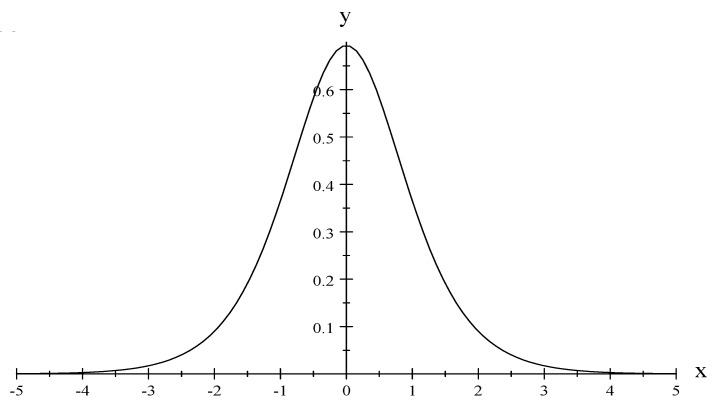
$y=\frac{S}{Nk}$ and x=βϵ. For T→0, S→0 and for T→∞, S→cte. Note that x>0.

**Figure 2:**
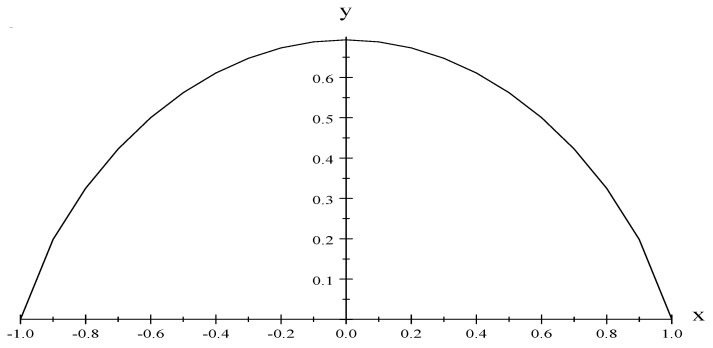
$y=\frac{S}{Nk}$ and $x=\frac{U}{\mu G}$. The entropy satisfies the third law of Thermodynamics. Note that when m>0 as it has to be considered, U<0.

G=NBthenumbermagneticfield,

where *G* is defined as an extensive special variable which will help to obtain an expression for the entropy as a fundamental equation; that is, S=S(U,G,N), if it exists, will represent a fundamental equation and, as a consequence, a thermodynamic system. In order to verify that Equation (73) represents the entropy of the paramagnetic solid, let us begin by taking the result derived by Greiner et al. [14] for j=1/2, and we have that (see Figure 1):

(78) $$S=Nk\left[\ln\left(2\cosh\beta \epsilon \right)-\beta \epsilon \tanh\beta \epsilon \right],$$

where ϵ=μB. Using Equation (70) in Equation (78), we obtain Equation (73). Introducing in Equation (73) *U*, *G* and *N*, and using the other equation of state,

(79) U=−MB=−mG,

where $m=\frac{M}{N}$, we have (see Figure 2),

(80) $$S=-\frac{Nk}{2}\left[\left(1+\frac{U}{\mu G}\right)\ln\left(1+\frac{U}{\mu G}\right)+\left(1-\frac{U}{\mu G}\right)\ln\left(1-\frac{U}{\mu G}\right)\right]+Nk\ln2.$$

This expression for the entropy is now in the form of a fundamental equation, S(U,G,N), and it can be used to directly obtain the TdS equation, the equations of state, and in particular the chemical potential ϕ.

Then,

(81) $$\frac{1}{T}=-\frac{Nk}{2}\left[\frac{1}{\mu G}\ln\left(1+\frac{U}{\mu G}\right)-\frac{1}{\mu G}\ln\left(1-\frac{U}{\mu G}\right)\right].$$

Using Equation (79), we obtain

(82) $$\begin{array}{c} \begin{array}{ccc} \frac{1}{T} & = & \frac{Nk}{\mu G}\frac{1}{2}\ln\left[\frac{\left(1+\frac{m}{\mu }\right)}{\left(1-\frac{m}{\mu }\right)}\right]\\ \frac{1}{T} & = & \frac{Nk}{\mu G}\tanh^{-1}\frac{m}{\mu }\\ \frac{m}{\mu } & = & \tanh\frac{\mu G}{NkT}\Rightarrow M=\mu N\tanh\frac{\mu B}{kT}, \end{array} \end{array}$$

which represents the equation of state as expected, Equation (72). Note that if *m* is negative, we have a negative temperature. For now, we consider m>0, and we postpone the discussion of the possibility of having m<0 below in this subsection. Further,

(83) $$\begin{array}{ccc} {\left[\frac{\partial S}{\partial G}\right]}_{U,N} & = & -\frac{UNk}{\mu G^{2}}\left[\frac{1}{2}\ln\left(\frac{1+\frac{m}{\mu }}{1-\frac{m}{\mu }}\right)\right]\\ & = & -\frac{UNk}{\mu G^{2}}\tanh^{-1}\frac{m}{\mu }=\frac{mk}{\mu B}\tanh^{-1}\frac{m}{\mu }. \end{array}$$

Using Equation (82), we arrive at

(84) $${\left[\frac{\partial S}{\partial G}\right]}_{U,N}=\frac{mk}{\mu B}\tanh^{-1}\frac{m}{\mu }=\frac{mk}{\mu B}\tanh^{-1}\left[\tanh\frac{\mu B}{kT}\right]=\frac{m}{T}.$$

Then,

(85) $$\begin{array}{ccc} {\left[\frac{\partial S}{\partial N}\right]}_{U,G} & = & -\frac{k}{2}\left[\left(1+\frac{U}{\mu G}\right)\ln\left(1+\frac{U}{\mu G}\right)+\left(1-\frac{U}{\mu G}\right)\ln\left(1-\frac{U}{\mu G}\right)\right]+k\ln2\\ & = & -\frac{\phi }{T}=\frac{S}{N} \end{array}$$

We can now express dS as

(86) $$dS=\frac{1}{T}dU+{\left[\frac{\partial S}{\partial G}\right]}_{U,N}dG+{\left[\frac{\partial S}{\partial N}\right]}_{U,G}dN.$$

Finally, the TdS equation can be written as

(87) $$TdS=dU+mdG+\frac{ST}{N}dN.$$

On the other hand,

(88) $$dU=TdS-mdG-\frac{ST}{N}dN.$$

If we consider the case where *N* is constant, we arrive at

(89) dU=TdS−md(NG)=dU=TdS−MdB,

which coincides with Equation (76) obtained by Barrett and Macdonald [13].

Finally, we have shown how to construct a fundamental equation for the paramagnetic solid with the *N* variable. It has to be highlighted that when *N* is not constant and we want to represent the TdS equation in the representation *U*, *B* and *N*, we arrive at

(90) $$\begin{array}{ccc} TdS & = & dU+mdG+\frac{ST}{N}dN\\ & = & dU+MdB+\left(\frac{MB+ST}{N}\right)dN. \end{array}$$

Note that $\left(\frac{MB+ST}{N}\right)/T$ represents ${\left(\partial S/\partial N\right)}_{U,B}$ and not minus the chemical potential (−ϕ).

#### 4.4.1. Hessian Method

In order to calculate the Hessian, we need to express U=U(S,G,N). However, an inspection of Equation (80) shows that it is not possible to write explicitly $U=U(S,\hat{H},N)$, and consequently, it is not possible to directly obtain the Hessian. In Appendix C, the Hessian for the paramagnetic solid is calculated. Since we have all the entries for the Hessian and by putting $\rho =\frac{\mu G}{NkT}$ and $\psi =\ln\left(2\cosh\rho \right)$, the characteristic polynomial is:

(91) $$\left(\begin{array}{c} T^{2}\left(\frac{N^{2}+G^{2}}{N^{2}G^{2}}\right)\\ +{\left(\frac{\mu }{N\rho }\psi -\frac{\mu }{N}\tanh\rho \right)}^{2} \end{array}\right)\lambda +\left(\begin{array}{c} \frac{T}{kN\rho^{2}}\cosh^{2}\rho +\frac{2kT}{N}\psi -\frac{2\mu G}{N^{2}}\tanh\rho \\ +{\left(\frac{k^{\frac{1}{2}}T^{\frac{1}{2}}}{N^{\frac{1}{2}}\rho }\cosh\rho \psi -\frac{k^{\frac{1}{2}}T^{\frac{1}{2}}}{N^{\frac{1}{2}}}\sinh\rho \right)}^{2} \end{array}\right)\lambda^{2}-\lambda^{3}=0.$$

We can see that one of the eigenvalues is λ=0 as it is required, and the other two are

$$\lambda_{\pm }=\alpha \pm \beta ,$$

with

$$\alpha =\frac{1}{2}\left(\begin{array}{c} \frac{T}{kN\rho^{2}}\cosh^{2}\rho +\frac{2kT}{N}\psi -\frac{2\mu G}{N^{2}}\tanh\rho \\ +{\left(\frac{k^{\frac{1}{2}}T^{\frac{1}{2}}\psi }{N^{\frac{1}{2}}\rho }\cosh\rho -\frac{k^{\frac{1}{2}}T^{\frac{1}{2}}}{N^{\frac{1}{2}}}\sinh\rho \right)}^{2} \end{array}\right)$$

and

$$\beta =\frac{1}{2}\left[\begin{array}{c} {\left(\begin{array}{c} \frac{T}{kN\rho^{2}}\cosh^{2}\rho +\frac{2kT\psi }{N}-\frac{2\mu G}{N^{2}}\tanh\rho \\ +{\left(\frac{k^{\frac{1}{2}}T^{\frac{1}{2}}\psi }{N^{\frac{1}{2}}\rho }\cosh\rho -\frac{k^{\frac{1}{2}}T^{\frac{1}{2}}}{N^{\frac{1}{2}}}\sinh\rho \right)}^{2} \end{array}\right)}^{2}+\left(\begin{array}{c} 4T^{2}\left(\frac{N^{2}+G^{2}}{N^{2}G^{2}}\right)\\ +4{\left(\frac{\mu \psi }{N\rho }-\frac{\mu }{N}\tanh\rho \right)}^{2} \end{array}\right) \end{array}\right]$$

Therefore, the system satisfies the thermodynamic requirements.

#### 4.4.2. Entropy Method

We have already proven item **I**, that is, the entropy is a first-degree homogeneous function (80), and items **A** and **G** are satisfied by looking at Equations (79), (82) and (80). Therefore, we just need to prove conditions **H** (∂S/∂U>0) and **G** (S>0) are satisfied, that is, for condition **H**, we have shown in the Hessian that λ are positive if *m* is positive, which is equivalent to T>0 [5]. However, from a statistical point of view, it is possible to have a negative temperature which comes from an initial oriented magnetization due to use a contrary direction of the magnetic field and suddenly changes the direction of it. However, this represents a non-equilibrium system [14,15]. Therefore, we can consider that the entropy is positive in a regular situation with *m* positive (m>0→S>0). For condition **G**, it is clear that all the first and second derivatives exist. Further, note that obtaining Equation (73) is left as an exercise (Problem 2.4) in Reif’s book [16] and the discussion of negative temperatures can be found in Problem (3.2).

#### 4.4.3. Completeness Method

In general, we can express the TdS equations of this system using Equation (15). In this case, we know that we only need three linearly independent equations. Thus, we choose the 1-equation, the 2-equation, and the 5-equation. That is, in these three equations, all the differentials of the variables *T*, *m*, ϕ, $\hat{H}$, and *N* are included in one of the equations as it is required to obtain the three independent equations.

The 1-equation is:

(92) $$TdS=a_{0}^{1}dT+a_{1}^{1}dm+A_{1}^{1}dG,$$

From Equations (15) and (16), we need to know $U=U\left(T,m,G\right)$, $N=N\left(T,m,G\right)$, and $\phi =\phi \left(T,m,G\right)$, that is:

$$\begin{array}{ccc} U & = & -mG,\\ N & = & \frac{-2G\mu }{Tk\left[\ln\left(1-\frac{m}{\mu }\right)-\ln\left(1+\frac{m}{\mu }\right)\right]},\\ \phi & = & \frac{Tk}{2}\left[\left(1-\frac{m}{\mu }\right)\ln\left(1-\frac{m}{\mu }\right)+\left(1+\frac{m}{\mu }\right)\ln\left(1+\frac{m}{\mu }\right)-2\ln2\right]. \end{array}$$

Hence,

$$\begin{array}{ccc} a_{0}^{1} & = & C_{m,G}=-\frac{\mu G}{T\ln\left(\frac{\mu -m}{\mu +m}\right)}\left[\begin{array}{c} \left(1-\frac{m}{\mu }\right)\ln\left(1-\frac{m}{\mu }\right)\\ +\left(1+\frac{m}{\mu }\right)\ln\left(1+\frac{m}{\mu }\right)-2\ln2 \end{array}\right],\\ a_{1}^{1} & = & -G+\frac{G\left(\frac{2\mu^{2}}{\mu^{2}-m^{2}}\right)}{\ln^{2}\left(\frac{\mu -m}{\mu +m}\right)}\left[\begin{array}{c} \left(1-\frac{m}{\mu }\right)\ln\left(1-\frac{m}{\mu }\right)\\ +\left(1+\frac{m}{\mu }\right)\ln\left(1+\frac{m}{\mu }\right)-2\ln2 \end{array}\right],\\ A_{1}^{1} & = & \frac{\mu }{\ln\left(\frac{\mu -m}{\mu +m}\right)}\left[\begin{array}{c} \left(1-\frac{m}{\mu }\right)\ln\left(1-\frac{m}{\mu }\right)\\ +\left(1+\frac{m}{\mu }\right)\ln\left(1+\frac{m}{\mu }\right)-2\ln2 \end{array}\right]. \end{array}$$

The 2-equation is

(93) $$TdS=a_{0}^{2}dT+a_{1}^{2}dm+A_{2}^{2}dN,$$

From Equations (15) and (16), we need to know $U=U\left(T,m,N\right)$, $G=G\left(T,m,N\right)$, and $\phi =\phi \left(T,m,N\right)$, that is:

$$\begin{array}{ccc} U & = & m\frac{kTN}{2\mu }\left[\ln\left(1-\frac{m}{\mu }\right)-\ln\left(1+\frac{m}{\mu }\right)\right],\\ G & = & -\frac{kTN}{2\mu }\left[\ln\left(1-\frac{m}{\mu }\right)-\ln\left(1+\frac{m}{\mu }\right)\right],\\ \phi & = & \frac{Tk}{2}\left[\left(1-\frac{m}{\mu }\right)\ln\left(1-\frac{m}{\mu }\right)+\left(1+\frac{m}{\mu }\right)\ln\left(1+\frac{m}{\mu }\right)-2\ln2\right] \end{array}$$

Therefore,

$$\begin{array}{ccc} a_{0}^{2} & = & 0,\\ a_{1}^{2} & = & \frac{kTN}{2\mu }\ln\left(\frac{\mu -m}{\mu +m}\right),\\ A_{2}^{2} & = & -\frac{kT}{2}\left[\begin{array}{c} \left(1-\frac{m}{\mu }\right)\ln\left(1-\frac{m}{\mu }\right)\\ +\left(1+\frac{m}{\mu }\right)\ln\left(1+\frac{m}{\mu }\right)-2\ln2 \end{array}\right]. \end{array}$$

The 5-equation is

(94) $$TdS=a_{1}^{5}dm+a_{2}^{5}d\phi +A_{1}^{5}dG,$$

From Equations (15) and (16), we need to know $U=U\left(m,\phi ,G\right)$ and $N=N\left(m,\phi ,G\right)$. We have

U=−mG,

$$N=\frac{-G\mu }{\phi \left[\ln\left(1-\frac{m}{\mu }\right)-\ln\left(1+\frac{m}{\mu }\right)\right]}\left[\left(1-\frac{m}{\mu }\right)\ln\left(1-\frac{m}{\mu }\right)+\left(1+\frac{m}{\mu }\right)\ln\left(1+\frac{m}{\mu }\right)\right],$$

and

$$T=\frac{2\phi }{k\left[\left(1-\frac{m}{\mu }\right)\ln\left(1-\frac{m}{\mu }\right)+\left(1+\frac{m}{\mu }\right)\ln\left(1+\frac{m}{\mu }\right)\right]},$$

$$\frac{1}{T}=\frac{k\left[\left(1-\frac{m}{\mu }\right)\ln\left(1-\frac{m}{\mu }\right)+\left(1+\frac{m}{\mu }\right)\ln\left(1+\frac{m}{\mu }\right)\right]}{2\phi },$$

Then, by putting $\rho =\begin{array}{c} \left(1-\frac{m}{\mu }\right)\ln\left(1-\frac{m}{\mu }\right)+\left(1+\frac{m}{\mu }\right)\ln\left(1+\frac{m}{\mu }\right)-2\ln2 \end{array}$ and $\psi =\left[\ln\left(1-\frac{m}{\mu }\right)-\ln\left(1+\frac{m}{\mu }\right)\right]$, we obtain

(95) $$a_{1}^{5}=-2G+\frac{2G\mu^{2}\rho }{\left(\mu -m\right)\left(\mu +m\right)\psi^{2}},\;a_{2}^{5}=-\frac{G\mu \rho }{\phi \psi }\;and\;A_{1}^{5}=\frac{\mu \rho }{\psi }$$

##### Thermodynamic Identities

With the three linearly independent TdS equations, it is possible to calculate the rest of the TdS equations and in this way obtain different thermodynamic identities. In this case, it is possible from the 1-equation and the 2-equation to deduce the 7-equation, which can be calculated using the method previously described in Section 3, that is:

(96) $$TdS=a_{0}^{7}dT+A_{1}^{7}dG+A_{2}^{7}dN.$$

We have

(97) $$a_{0}^{7}=\frac{a_{1}^{2}a_{0}^{1}-a_{1}^{1}a_{0}^{2}}{a_{1}^{2}-a_{1}^{1}},\;A_{1}^{7}=\frac{A_{1}^{1}a_{1}^{2}}{a_{1}^{2}-a_{1}^{1}}\;\mathrm{and}\;A_{2}^{7}=-\frac{A_{2}^{2}a_{1}^{1}}{a_{1}^{2}-a_{1}^{1}}.$$

Then, by putting $a_{0}^{7}={\left(\frac{\partial U}{\partial T}\right)}_{G,N}=C_{G,N}$, and remembering that $a_{0}^{2}=0$, we have

$$C_{G,N}=\frac{a_{1}^{2}C_{m,G}}{a_{1}^{2}-a_{1}^{1}}.$$

We obtain

$$\left(C_{G,N}-C_{m,G}\right)=C_{G,N}\frac{a_{1}^{1}}{a_{1}^{2}}.$$

Or, in an equivalent form, we obtain a Mayer-like relation:

(98) $$\left(C_{G,N}-C_{m,G}\right)=C_{G,N}\frac{G\mu }{kTN\psi }\left(\frac{\left(\frac{4}{1-{\frac{m}{\mu^{2}}}^{2}}\right)}{\psi^{2}}\rho -2\right)$$

### 4.5. The Kelly Plasma Equation

For more than 70 years, there has been much interest in plasma physics due to its applications in Tokamaks and Astrophysics. However, under nonrelativistic conditions, the equations of state for an ideal gas state are used as the first approximation to calculate the equations of balance, the moment equations, and thermodynamic flows [17,18]. Nevertheless, among other proposals, Kelly [7] gave an equation of state for a plasma with different species relating the pressure *P*, the number of particles for each species $N_{i}$, the volume *V*, and the temperature *T*. However, one equation of state is not enough to describe a system composed of many species without giving another equation of state. This point was partially corrected by Wergeland [19] by including an expression for the corrected internal energy due to Debye and Hückel theory, where the energy is obtained by making an average of the Coulomb energy.

Our purpose in this subsection consists of using our method to complete the set of equations, as we did in Section 4.2 (Unconventional System: A Particular Case) and in Section 4.3 (The Rubber Band), obtaining the Debye–Hückel–Wergeland corrected total internal energy. Since in many situations in plasma physics, it is just necessary to consider only the electrons, we analyze the Kelly equations for one species. We obtain the entropy for a plasma composed of electrons where the effect of the ions is considered just in the equations of state. We obtain the entropy as a function of the volume *V*, the number of electrons *N*, and the temperature *T*. Since it is not possible to explicitly write the entropy as a function of the internal energy *U*, the volume *V*, and the number of electrons *N*, that is, the entropy as a fundamental equation, we develop a method to obtain the Hessian in order to check the viability of the system similar to what we have done in Section 4.2 (Unconventional System: A Particular Case). Indeed, in plasma physics, it is very important to know the relaxation times which are related with the eigenvalues of the Hessian. We also analyze the first-degree homogeneous property of the entropy using a technique exposed in Section 2.4. We also show that the temperature *T* is positive. The TdS equations can be obtained from the results obtained for calculating the Hessian.

The Kelly equation of state for a system with different species is:

(99) $$P=\sum_{\alpha }\frac{N_{\alpha }}{V}kT\left(1-\frac{1}{18N_{D}}\right).$$

If we consider just one species (electrons) but keeping the interaction with the ions of the system, we have

(100) $$P=\frac{N}{V}kT\left(1-\frac{1}{18N_{D}}\right)=\frac{N}{V}kT\left(1-\frac{1}{24\frac{N}{V}{\left(\frac{VkT}{4\pi Ne^{2}}\right)}^{\frac{3}{2}}}\right),$$

where

(101) $$N_{D}=\frac{4}{3}\pi D^{3}\frac{N}{V}\;D={\left(\frac{k}{4\pi e^{2}Z_{1}^{2}}\right)}^{\frac{1}{2}}\frac{V^{\frac{1}{2}}T^{\frac{1}{2}}}{N^{\frac{1}{2}}}={\left(\frac{kTV}{4\pi Ne^{2}}\right)}^{1/2}.$$

It is necessary to note that *D* is Debye’s length and $N_{D}$ represents the number of particles contained in Debye’s sphere. The contribution of the pressure that corrects the ideal gas is

(102) $$P_{corr}=-\frac{N}{V}kT\frac{1}{24\pi \frac{N}{V}{\left(\frac{VkT}{4\pi Ne^{2}}\right)}^{\frac{3}{2}}}=-\frac{1}{24\pi }\frac{kT}{D^{3}}.$$

Following Wergeland [19], the contribution of the energy should be

(103) $$U_{corr}=-\frac{1}{2}N\frac{e^{2}}{D}.$$

We note that

(104) $$P_{corr}=\frac{1}{3}\frac{U_{corr}}{V},$$

which has the form of the equation of state for bosons and fermions. Let us analyze Equation (104); we have

(105) $$\begin{array}{ccc} \frac{1}{3}\frac{U_{corr}}{V} & = & \frac{1}{3}\left(-\frac{1}{2}N\frac{e^{2}}{VD}\right)=-\frac{1}{6}\frac{Ne^{2}}{VD}\\ & = & -\frac{1}{6}\frac{Ne^{2}}{V{\left(\frac{kTV}{4\pi Ne^{2}}\right)}^{1/2}}=-\frac{1}{6\times 4\pi }\frac{kT}{{\left(\frac{k^{3}T^{3}V^{3}}{4^{3}\pi^{3}N^{3}e^{6}}\right)}^{1/2}}=-\frac{1}{24\pi }\frac{kT}{D^{3}}. \end{array}$$

Finally, we can write the total pressure as

(106) P=NkTV−NVkT124πNVVkT4πNe232  orP=NkTV−N323V32T12π12e3k12,

and the total energy as

(107) U=32NkT+3VPcorr  orU=32NkT−N32V12T12π12e3k12.

It is interesting that we can obtain the heat capacity $C_{V,N}$, that is:

(108) $$C_{V,N}={\left[\frac{\partial U}{\partial T}\right]}_{V,N}=\frac{3}{2}Nk+\frac{N^{\frac{3}{2}}}{2V^{\frac{1}{2}}T^{\frac{3}{2}}}\left(\frac{\pi^{\frac{1}{2}}e^{3}}{k^{\frac{1}{2}}}\right).$$

Due to the similarity with the relationship between energy and pressure in the case of photons, we can propose a correction for the entropy given by the following expression

(109) $$S_{corr}=\frac{U_{corr}}{3T}.$$

Note that due to the expression of the corrected energy in our case (see Equation (107)), there is a factor 1/3 and not a factor 4/3 as it happens in the photon case. Thus, we can propose the total entropy as

(110) $$S=S_{ig}+S_{corr},$$

where $S_{ig}$ represents the entropy for a null charge, e=0, that is, the ideal gas. It has to be noted that although $S_{ig}$ is formally the entropy of the ideal gas, it cannot be substituted in another functionality, that is, the expression is good for our purpose only when it is a function of the volume *V*, the temperature *T*, and the number of particles *N*. Therefore,

(111) $$\begin{array}{ccc} S & = & S_{ig}+\frac{U_{corr}}{3T}\\ & = & S_{ig}-\frac{N^{\frac{3}{2}}}{3V^{\frac{1}{2}}T^{\frac{3}{2}}}\left(\frac{\pi^{\frac{1}{2}}e^{3}}{k^{\frac{1}{2}}}\right). \end{array}$$

In order to verify that this proposed entropy describes the system, it is necessary to check that we can obtain the equations of state. For this purpose, let us calculate the Helmholtz free energy *A*, that is:

(112) $$\begin{array}{ccc} A & = & U-TS\\ & = & U_{ig}+U_{corr}-T\left(S_{ig}+S_{corr}\right)\\ A & = & A_{ig}+U_{corr}-TS_{corr}. \end{array}$$

Now, we can calculate the pressure *P*,

(113) $$\begin{array}{ccc} P & = & -{\left[\frac{\partial A}{\partial V}\right]}_{T,N}=\frac{NkT}{V}-\left[\frac{\partial U_{corr}}{\partial V}\right]+T\left[\frac{\partial S_{corr}}{\partial V}\right]\\ & = & \frac{NkT}{V}-\left[\frac{N^{\frac{3}{2}}}{2V^{\frac{3}{2}}T^{\frac{1}{2}}}\left(\frac{\pi^{\frac{1}{2}}e^{3}}{k^{\frac{1}{2}}}\right)\right]+T\left[\frac{N^{\frac{3}{2}}}{6V^{\frac{3}{2}}T^{\frac{3}{2}}}\left(\frac{\pi^{\frac{1}{2}}e^{3}}{k^{\frac{1}{2}}}\right)\right]\\ & = & \frac{NkT}{V}-\frac{N^{\frac{3}{2}}}{3V^{\frac{3}{2}}T^{\frac{1}{2}}}\left(\frac{\pi^{\frac{1}{2}}e^{3}}{k^{\frac{1}{2}}}\right), \end{array}$$

and the equation of state is verified. Using Equations (111) and (112), the equation of state for *U* can also be verified.

#### 4.5.1. Hessian Method

In order to calculate the Hessian, we need to express U=U(S,V,N). However, an inspection of Equation (111) shows that it is not possible to write explicitly U=U(S,V,N), and consequently, it is not possible to directly obtain the Hessian. In Appendix D, the Hessian for the Kelly plasma is calculated. Once the Hessian is obtained and by putting $\rho =12N^{2}T^{5}V^{3}$ and $\psi =T^{\frac{5}{2}}V^{\frac{5}{2}}$, the characteristic polynomial is:

(114) $$\begin{array}{c} \left(\begin{array}{c} +2\rho N^{2}{\left(kN+S_{corr}\right)}^{2}-2\rho k^{2}N^{2}{\left(S_{ig}+S_{corr}\right)}^{2}\\ -\rho kNS_{corr}{\left(S_{ig}+S_{corr}\right)}^{2}-N^{2}\rho {\left(S_{corr}+2kN\right)}^{2}\\ +{\left(kN^{2}\psi -6NS_{corr}\psi \right)}^{2}+3\rho {\left(S_{ig}S_{corr}+S_{corr}^{2}\right)}^{2}\\ +\left(2S_{corr}-kN\right)N^{2}S_{corr}\rho -25k^{2}N^{4}\psi^{2} \end{array}\right)\lambda \\ +\left(\begin{array}{c} 10N^{2}\psi V^{-2}{\left(kN-S_{corr}\right)}^{2}\\ +4\psi {\left(S_{ig}-kN\right)}^{2}+2\left(3k^{2}+2\right)N^{2}\psi \\ +\left(19kN-18S_{corr}\right)N^{2}S_{corr}\psi V^{-2}\\ +3\left(4S_{ig}-3kN\right)S_{corr}\psi \end{array}\right)\lambda^{2}-\lambda^{3}=0. \end{array}$$

Therefore, we found an eigenvalue λ=0, and the other two eigenvalues are:

(115) $$\lambda_{\pm }=\alpha \pm \beta ,$$

with

(116) $$\alpha =\frac{1}{2}\left(\begin{array}{c} 10N^{2}\psi V^{-2}{\left(kN-S_{corr}\right)}^{2}+4\psi {\left(S_{ig}-kN\right)}^{2}+2\left(3k^{2}+2\right)N^{2}\psi \\ +\left(19kN-18S_{corr}\right)N^{2}S_{corr}\psi V^{-2}+3\left(4S_{ig}-3kN\right)S_{corr}\psi \end{array}\right)$$

and

(117) $$\beta =\frac{1}{2}{\left[\begin{array}{c} {\left(\begin{array}{c} 10N^{2}\psi V^{-2}{\left(kN-S_{corr}\right)}^{2}\\ +4\psi {\left(S_{ig}-kN\right)}^{2}+2\left(3k^{2}+2\right)N^{2}\psi \\ +\left(19kN-18S_{corr}\right)N^{2}S_{corr}\psi V^{-2}\\ +3\left(4S_{ig}-3kN\right)S_{corr}\psi \end{array}\right)}^{2}\\ +4\left(\begin{array}{c} +2\rho N^{2}{\left(kN+S_{corr}\right)}^{2}-2\rho k^{2}N^{2}{\left(S_{ig}+S_{corr}\right)}^{2}\\ -\rho kNS_{corr}{\left(S_{ig}+S_{corr}\right)}^{2}-N^{2}\rho {\left(S_{corr}+2kN\right)}^{2}\\ +{\left(kN^{2}\psi -6NS_{corr}\psi \right)}^{2}+3\rho {\left(S_{ig}S_{corr}+S_{corr}^{2}\right)}^{2}\\ +\left(2S_{corr}-kN\right)N^{2}S_{corr}\rho -25k^{2}N^{4}\psi^{2} \end{array}\right) \end{array}\right]}^{\frac{1}{2}}.$$

#### 4.5.2. Entropy Method

The items **A**, **F**, and **G** can be verified by looking at Equations (106), (107) and (A32). Although we have not been able to deduce the entropy as a fundamental equation for this system, we can apply the technique developed in Section 2.4 in order to verify item **I**. Therefore, we need to prove Equation (A1) in our case (see Appendix A), that is:

$$\begin{array}{ccc} S(T,\lambda V,\lambda N) & = & \lambda S_{ig}-\frac{\lambda^{\frac{3}{2}}N^{\frac{3}{2}}}{3\lambda^{\frac{1}{2}}V^{\frac{1}{2}}T^{\frac{3}{2}}}\left(\frac{\pi^{\frac{1}{2}}e^{3}}{k^{\frac{1}{2}}}\right)\\ & = & \lambda \left(S_{ig}-\frac{N^{\frac{3}{2}}}{3V^{\frac{1}{2}}T^{\frac{3}{2}}}\left(\frac{\pi^{\frac{1}{2}}e^{3}}{k^{\frac{1}{2}}}\right)\right)\\ & = & \lambda S(T,V,N) \end{array}$$

It is necessary to prove that the temperature is positive (item **H**). We know from Equations (102), (103) and (104) that:

$$U_{corr}=-\frac{1}{2}N\frac{e^{2}}{D},\;P_{corr}=-\frac{1}{24\pi }\frac{kT}{D^{3}},\;\mathrm{and}\;P_{corr}=\frac{1}{3}\frac{U_{corr}}{V},$$

which implies that T>0.

On the other hand, due to the fact that that the number of particles contained in a Debye sphere is big, the isothermal compressibility is positive,

$$\kappa_{T}=-\frac{1}{V}{\left(\frac{\partial V}{\partial P}\right)}_{T,N}=\frac{1}{kT}{\left(\frac{N}{V}-\frac{1}{16}\frac{1}{{\left(\frac{VkT}{4\pi Ne^{2}}\right)}^{\frac{3}{2}}}\right)}^{-1}>0$$

Therefore, the Kelly completed plasma represents a stable system.

## 5. Concluding Remarks

In the present work, we have compared two different methods to study the viability of a system represented by a set of equations and developed a method to give a complete view of a thermodynamic system. We arrive to the following comments for each one.

**The Hessian Method**: The principal method consists of obtaining a characteristic polynomial, one of its eigenvalues being null. The other eigenvalues must be real positive values which are related with the relaxation times. This last property is possibly the most important aspect of this method.

**The Entropy Method**: The method consists of noting that the first-degree homogeneous entropy must comply with ${\left[\partial U/\partial S\right]}_{\left\{X^{n}\right\}}>0$ or ${\left[\partial S/\partial U\right]}_{\left\{X^{n}\right\}}>0$. This method is the simplest one.

**Completeness Method**: This method provides as much information as possible of a thermodynamic system. It permits knowing all the TdS equations and obtaining the thermodynamic identities.

**General Results**:

(*i*) We have shown how to obtain a complete set of equations from an incomplete set of equations.
(*ii*) We have proven that by applying the Gibbs–Duhem method, a first-degree homogeneous entropy is obtained. That is, Boltzmann counting is included in the method.
(*iii*) We have developed a method to obtain the Hessian of a thermodynamic system without knowing the expression of the fundamental equation in the energy picture.
(*iv*) We have developed a method to correct a TdS equation when this last one is not well-defined.
(*v*) Novel thermodynamic identities have been found for each analyzed system.
(*vi*) These three methods have been applied to the ideal gas, to an unconventional system, to the rubber band, to the paramagnetic solid, and to the Kelly plasma, but they can be applied to analyze any thermodynamic system that presents a problem in order to correct it or to obtain all the information which can be derived from it.

## Appendix A

The purpose of this appendix is to give a method that allows us to demonstrate when the entropy is a first-degree homogeneous function if we do not know *S* as a fundamental equation. That is, in the case of having proven the items **A, G**, and **H** without having been able to deduce the entropy as a fundamental equation for this system, for example, if we do not know S=S(U,V,N) and we know S=S(T,V,N), it is possible to check the first-degree homogeneous property of the entropy using the below result. Let us suppose Equation (2) obeys this property, then we have

λS=S(λU,λV,λN).

However if we know, for example, S=S(T,V,N), we have

$$S\left(U,V,N\right)=S\left(T,V,N\right)=S\left({\left(\frac{\partial U(S,V,N)}{\partial S}\right)}_{V,N},V,N\right)$$

Then,

S(λU,λV,λN)=λS(U,V,N)=λS(T,V,N).

On the other hand,

$$\begin{array}{ccc} S(\lambda U,\lambda V,\lambda N) & = & S\left(\left(\frac{\partial U(\lambda S,\lambda V,\lambda N)}{\partial \lambda S}\right),\lambda V,\lambda N)\right)\\ & = & S(T,\lambda V,\lambda N). \end{array}$$

Therefore, it is necessary to prove that

(A1) S(T,λV,λN)=λS(T,V,N).

That is, we can obviously generalize the result to any representation of the entropy; that is, if

(A2) $$S\left(\left\{x_{l}\right\},\left\{\lambda X^{j}\right\},\right)=\lambda S\left(\left\{x_{l}\right\},\left\{X^{j}\right\}\right),$$

where $x_{l}$ and $X^{k}$ are intensive and extensive variables, respectively, such that the total number of them is n+1, and the entropy is a first-degree homogeneous function in the extensive variables. This result is analyzed in the Kelly Plasma case, Section 4.5.2.

## Appendix B

Let us calculate some of the TdS equations for the ideal gas with the *N* variable.

The 2-equation is:

Let us calculate the 2-, 3-, 4-, and 9-equations for the ideal gas with the *N* variable.

(A3) $$TdS=a_{0}^{2}dT+a_{1}^{2}dP+A_{2}^{2}dN,$$

where from Equation (16),

(A4) $$\begin{array}{ccc} a_{0}^{2} & = & {\left(\frac{\partial U}{\partial T}\right)}_{P,N}+P{\left(\frac{\partial V}{\partial T}\right)}_{P,N},\;a_{1}^{2}={\left(\frac{\partial U}{\partial P}\right)}_{T,N}+P{\left(\frac{\partial V}{\partial P}\right)}_{T,N},\\ A_{2}^{2} & = & {\left(\frac{\partial U}{\partial N}\right)}_{T,P}-\mu +P{\left(\frac{\partial V}{\partial N}\right)}_{T,P}.A4 \end{array}$$

In order to calculate the above relations, we need to know the internal energy and the volume as functions of *T*, *P*, and *N*, that is, U=U(T,P,N) and V=V(T,P,N). Hence,

(A5) $$U=\frac{3}{2}NkT\;\mathrm{and}\;V=\frac{NkT}{P}.$$

Consequently, the 2-equation is

(A6) $$TdS=\frac{5}{2}NkdT-\frac{NkT}{P}dP+\left[\frac{5}{2}kT-\mu \right]dN.$$

The 3-equation is:

(A7) $$TdS=a_{0}^{3}dT+a_{2}^{3}d\mu +A_{1}^{3}dV,$$

where

(A8) $$\begin{array}{ccc} a_{0}^{3} & = & {\left(\frac{\partial U}{\partial T}\right)}_{\mu ,V}-\mu {\left(\frac{\partial N}{\partial T}\right)}_{\mu ,V},\;a_{2}^{3}={\left(\frac{\partial U}{\partial \mu }\right)}_{T,V}-\mu {\left(\frac{\partial N}{\partial \mu }\right)}_{T,V},\\ A_{1}^{3} & = & {\left(\frac{\partial U}{\partial V}\right)}_{T,\mu }+P-\mu {\left(\frac{\partial N}{\partial V}\right)}_{T,\mu }. \end{array}$$

From Equations (15) and (16) for this case, we need to know U=U(T,μ,V) and N=N(T,μ,V). We use

(A9) $$N=T^{\frac{3}{2}}{\left(\frac{3k}{2U_{0}}\right)}^{\frac{3}{2}}\left(\frac{V}{V_{0}}\right){\left(\frac{1}{N_{0}}\right)}^{-\frac{5}{2}}e^{\left\{\frac{\mu }{kT}+\frac{s_{0}}{k}-\frac{5}{2}\right\}},$$

(A10) $$U=\frac{3}{2}RT^{\frac{5}{2}}{\left(\frac{3k}{2U_{0}}\right)}^{\frac{3}{2}}\left(\frac{V}{V_{0}}\right){\left(\frac{1}{N_{0}}\right)}^{-\frac{5}{2}}e^{\left\{\frac{\mu }{kT}+\frac{s_{0}}{k}-\frac{5}{2}\right\}},$$

Therefore, the 3-equation is

(A11) $$TdS=N\left[\frac{15}{2}k-\frac{3\mu }{T}+\frac{\mu^{2}}{kT^{2}}\right]dT+N\left[\frac{3}{2}-\frac{\mu }{kT}\right]d\mu +\frac{N}{V}\left[\frac{5}{2}kT-\mu \right]dV.$$

The 4-equation is:

(A12) $$TdS=a_{0}^{4}dT+a_{2}^{4}d\mu +A_{2}^{4}dN,$$

where

(A13) $$\begin{array}{ccc} a_{0}^{4} & = & {\left(\frac{\partial U}{\partial T}\right)}_{\mu ,N}+P{\left(\frac{\partial V}{\partial T}\right)}_{\mu ,N},\;a_{2}^{4}={\left(\frac{\partial U}{\partial \mu }\right)}_{T,N}+P{\left(\frac{\partial V}{\partial \mu }\right)}_{T,N},\\ A_{2}^{4} & = & {\left(\frac{\partial U}{\partial N}\right)}_{T,\mu }-\mu +P{\left(\frac{\partial V}{\partial N}\right)}_{T,\mu }. \end{array}$$

From Equations (15) and (16) for this case, we need to know U=U(T,μ,N) and V=V(T,μ,N). Hence,

(A14) $$U=\frac{3}{2}NkT,\;V={\left(\frac{2U_{0}}{3k}\right)}^{\frac{3}{2}}N_{0}^{-\frac{5}{2}}V_{0}NT^{-\frac{3}{2}}e\left\{\frac{5}{2}-\frac{s_{0}}{k}-\frac{\mu }{kT}\right\}.$$

Consequently, the 4-equation is

(A15) $$TdS=\frac{N\mu }{T}dT-Nd\mu +\left[\frac{5}{2}kT-\mu \right]dN.$$

The 9-equation is:

We can repeat the same procedure for the 9-equation

(A16) $$TdS=a_{2}^{9}d\mu +A_{1}^{9}dV+A_{2}^{9}dN$$

From Equations (15) and (16) for this case, we need to know $U=U\left(\mu ,V,N\right)$ and $P=P\left(\mu ,V,N\right)$ and μ. We have

(A17) $$\begin{array}{ccc} PV & = & NkT,\;U=\frac{3}{2}NkT,\\ \mu & = & T\left[-\frac{3}{2}k\ln\left(\frac{U}{Nu_{0}}\right)-k\ln\left(\frac{V}{Nv_{0}}\right)+{\left(\frac{\mu }{T}\right)}_{0}\right]. \end{array}$$

We solve for *U* and *P* and obtain

(A18) $$U=-\frac{\mu N}{W\left(-\frac{\mu {\left(\frac{V}{Nv_{0}}\right)}^{\frac{2}{3}}\exp\left(-\frac{2{\left(\frac{\mu }{T}\right)}_{0}}{3k}\right)}{u_{0}}\right)},\;P=-\frac{2N\mu }{3VW\left(-\frac{\mu {\left(\frac{V}{Nv_{0}}\right)}^{\frac{2}{3}}\exp\left(-\frac{2{\left(\frac{\mu }{T}\right)}_{0}}{3k}\right)}{u_{0}}\right)}$$

where *W* is the Lambert function. Finally,

$$a_{2}^{9}=-\frac{N}{W(Z)}+\frac{N}{\left(1+W(Z)\right)W\left(Z\right)},\;A_{1}^{9}=\frac{2\mu N}{3V}\left(-\frac{1}{W(Z)}+\frac{1}{W\left(Z\right)+W^{2}\left(Z\right)}\right),$$

(A19) $$A_{2}^{9}=\frac{1}{3}\mu \left(-3-\frac{3}{W(Z)}-\frac{2}{W\left(Z\right)+W^{2}\left(Z\right)}\right),$$

where *W* is the solution along the real axis of the Lambert *W*-function, and we have put

$$Z=-\frac{\mu {\left(\frac{V}{Nv_{0}}\right)}^{\frac{2}{3}}\exp\left(-\frac{2{\left(\frac{\mu }{T}\right)}_{0}}{3k}\right)}{u_{0}}.$$

Finally, the 9-equation is

(A20) $$\begin{array}{ccc} TdS & = & \left[-\frac{N}{W(Z)}+\frac{N}{\left(1+W(Z)\right)W\left(Z\right)}\right]d\mu +\left[\frac{2\mu N}{3V}\left(-\frac{1}{W(Z)}+\frac{1}{W\left(Z\right)+W^{2}\left(Z\right)}\right)\right]dV\\ & & +\left[\frac{1}{3}\mu \left(-3-\frac{3}{W(Z)}-\frac{2}{W\left(Z\right)+W^{2}\left(Z\right)}\right)\right]dN. \end{array}$$

## Appendix C

In order to calculate the Hessian for the paramagnetic case, we need to express U=U(S,G,N). However, an inspection of Equation (80) shows that it is not possible to write explicitly $U=U(S,\hat{H},N)$, and consequently, it is not possible to directly obtain the Hessian. However, since the Hessian is constituted by entries of this kind

$${\left[\frac{\partial^{2}U}{\partial S\partial S}\right]}_{G,N}={\left[\frac{\partial T}{\partial S}\right]}_{G,N},$$

if we have an expression of T=T(S,G,N), it will be sufficient for obtaining this entry. That is, starting from Equations (78) and (79) and using ϵ=μB, we have

$$\begin{array}{ccc} \frac{S}{Nk} & = & \ln\left(\cosh\frac{\mu G}{kTN}\right)-\beta \epsilon \tanh\frac{\mu G}{kTN}+\ln2.\\ \frac{1}{Nk}{\left[\frac{\partial S}{\partial T}\right]}_{G,N} & = & \frac{{\left(\frac{\mu G}{kN}\right)}^{2}}{T^{3}}\frac{1}{\cosh^{2}\frac{\mu G}{NkT}}. \end{array}$$

That is

(A21) $${\left[\frac{\partial^{2}U}{\partial S\partial S}\right]}_{G,N}={\left[\frac{\partial T}{\partial S}\right]}_{G,N}=\frac{1}{{\left[\frac{\partial S}{\partial T}\right]}_{G,N}}=\frac{NkT^{3}}{\mu^{2}G^{2}}\cosh^{2}\frac{\mu G}{NkT}.$$

Then, for the entry

(A22) $${\left[\frac{\partial^{2}U}{\partial G\partial S}\right]}_{N}={\left[\frac{\partial T}{\partial G}\right]}_{S,N},$$

we can use

$${\left[\frac{\partial T}{\partial G}\right]}_{S,N}{\left[\frac{\partial G}{\partial S}\right]}_{T,N}{\left[\frac{\partial S}{\partial T}\right]}_{G,N}=-1.$$

That is

$${\left[\frac{\partial T}{\partial G}\right]}_{S,N}=-\frac{{\left[\frac{\partial S}{\partial G}\right]}_{T,N}}{{\left[\frac{\partial S}{\partial T}\right]}_{G,N}}.$$

Then, using the expression of the entropy and the temperature, Equations (80) and (81), we obtain

(A23) $$\frac{S}{Nk}=\ln\left(2\cosh\frac{\mu G}{kTN}\right)-\frac{\mu G}{kTN}\tanh\frac{\mu G}{kTN}$$

That is S=S(T,G,N) and we can calculate ${\left[\frac{\partial S}{\partial G}\right]}_{T,N}$ and ${\left[\frac{\partial S}{\partial T}\right]}_{G,N}$ in order to obtain ${\left[\frac{\partial^{2}U}{\partial G\partial S}\right]}_{N}$. Then,

$${\left[\frac{\partial S}{\partial G}\right]}_{T,N}=-\frac{\mu^{2}G{\mathrm{sech}}^{2}\frac{\mu G}{NkT}}{kNT^{2}}$$

Then

(A24) $${\left[\frac{\partial S}{\partial T}\right]}_{G,N}=\frac{\mu^{2}G^{2}{\mathrm{sech}}^{2}\frac{\mu G}{NkT}}{kNT^{3}}$$

Then,

(A25) $${\left[\frac{\partial^{2}U}{\partial G\partial S}\right]}_{N}={\left[\frac{\partial T}{\partial G}\right]}_{S,N}=-\frac{{\left[\frac{\partial S}{\partial G}\right]}_{T,N}}{{\left[\frac{\partial S}{\partial T}\right]}_{G,N}}=\frac{T}{G}.$$

Now, let us calculate

$${\left[\frac{\partial^{2}U}{\partial G\partial G}\right]}_{S,N}.$$

We need to know U=U(S,G,N). However, we know from Equation (88) that

$$\frac{\partial U}{\partial G}=-m.$$

Consequently, we need to know m=m(S,G,N) for obtaining

$${\left[\frac{\partial^{2}U}{\partial G\partial G}\right]}_{S,N}=-{\left[\frac{\partial m}{\partial G}\right]}_{S,N}.$$

However

$$\frac{U}{G}=-m,$$

then,

(A26) $$\begin{array}{ccc} {\left[\frac{\partial^{2}U}{\partial G\partial G}\right]}_{S,N} & = & -{\left[\frac{\partial \frac{U(S,G,N)}{G}}{\partial G}\right]}_{S,N}=\frac{U(S,G,N)}{G^{2}}-\frac{1}{G}{\left[\frac{\partial U}{\partial G}\right]}_{S,N}\\ & = & \frac{U(S,G,N)}{G^{2}}+\frac{m}{G}=\frac{-mG}{G^{2}}+\frac{m}{G}=0. \end{array}$$

Another term that we need to obtain for the Hessian is:

$${\left[\frac{\partial^{2}U}{\partial G\partial N}\right]}_{S}$$

We know that from Equation (88)

$${\left[\frac{\partial U}{\partial N}\right]}_{S,G}=-\frac{ST}{N}$$

Then, using Equation (A25)

(A27) $${\left[\frac{\partial^{2}U}{\partial G\partial N}\right]}_{S}={\left[\frac{\partial -\frac{ST}{N}}{\partial G}\right]}_{N,S}=-\frac{1}{N}{\left[\frac{\partial ST}{\partial G}\right]}_{N,S}=-\frac{S}{N}{\left[\frac{\partial T}{\partial G}\right]}_{N,S}=-\frac{S}{N}\frac{T}{G}.$$

We also need

$${\left[\frac{\partial^{2}U}{\partial S\partial N}\right]}_{G}.$$

We know from Equation (88) that

$${\left[\frac{\partial U}{\partial N}\right]}_{G,S}=-\frac{ST}{N}.$$

Using Equation (A24), we have

(A28) $$\begin{array}{ccc} {\left[\frac{\partial^{2}U}{\partial S\partial N}\right]}_{G} & = & {\left[-\frac{\partial \frac{ST}{N}}{\partial S}\right]}_{G,N}=-\frac{1}{N}{\left[\frac{\partial ST}{\partial S}\right]}_{G,N}=-\frac{1}{N}\left[T+S{\left[\frac{\partial T}{\partial S}\right]}_{G,N}\right]\\ & = & -\frac{T(S,G,N)}{N}-\frac{SkT^{3}}{\mu^{2}G^{2}}\cosh^{2}\frac{\mu G}{NkT}. \end{array}$$

Finally, we need to know

$${\left[\frac{\partial^{2}U}{\partial N\partial N}\right]}_{S,G}.$$

We know that from Equation (88)

$${\left[\frac{\partial U}{\partial N}\right]}_{S,G}=-\frac{ST}{N}.$$

Hence,

$${\left[\frac{\partial^{2}U}{\partial N\partial N}\right]}_{S,G}={\left[-\frac{\partial \frac{ST}{N}}{\partial N}\right]}_{S,G}=\frac{ST}{N^{2}}-\frac{S}{N}{\left[\frac{\partial T}{\partial N}\right]}_{S,G}.$$

We need to use

$${\left[\frac{\partial T}{\partial N}\right]}_{S,G}{\left[\frac{\partial N}{\partial S}\right]}_{T,G}{\left[\frac{\partial S}{\partial T}\right]}_{N,G}=-1$$

Accordingly, using Equations (A23) and (A24), we arrive at

$$\begin{array}{ccc} {\left[\frac{\partial T}{\partial N}\right]}_{S,G} & = & -{\left[\frac{\partial T}{\partial S}\right]}_{N,G}{\left[\frac{\partial S}{\partial N}\right]}_{T,G}\\ & = & -\frac{T}{N}-\frac{k^{2}NT^{3}}{\mu^{2}G^{2}{\mathrm{sech}}^{2}\frac{\mu G}{NkT}}\ln\left(2\cosh\frac{\mu G}{NkT}\right)+\frac{kT^{2}\cosh\frac{\mu G}{NkT}\sinh\frac{\mu G}{NkT}}{\mu G}. \end{array}$$

Therefore,

(A29) $$\begin{array}{ccc} {\left[\frac{\partial^{2}U}{\partial N\partial N}\right]}_{S,G} & = & {\left[-\frac{\partial \frac{ST}{N}}{\partial N}\right]}_{S,G}=\frac{2ST}{N^{2}}+\frac{Sk^{2}T^{3}}{\mu^{2}G^{2}{\mathrm{sech}}^{2}\frac{\mu G}{NkT}}\ln\left(2\cosh\frac{\mu G}{NkT}\right)\\ & & -\frac{SkT^{2}\cosh\frac{\mu G}{NkT}\sinh\frac{\mu G}{NkT}}{N\mu G}. \end{array}$$

## Appendix D

In order to calculate the Hessian for the Kelly plasma, we need to express U=U(S,V,N). However, an inspection of Equation (111) shows that it is not possible to write explicitly U=U(S,V,N), and consequently, it is not possible to directly obtain the Hessian. However, since the Hessian is constituted by entries of this kind,

(A30) $${\left[\frac{\partial^{2}U}{\partial S\partial S}\right]}_{V,N}={\left[\frac{\partial T}{\partial S}\right]}_{V,N},$$

Having an expression for T=T(S,V,N) will be sufficient. Then, let us give the expression of the entropy of the ideal gas using the Sackur–Tetrode expression:

(A31) $$S_{ig}=Nk\ln\left[\frac{VT^{\frac{3}{2}}}{N}\right]+N\left(\frac{5}{2}k+\frac{3}{2}k\ln\left[\frac{2k\pi m}{h^{2}}\right]\right)=Ns_{0}+Nk\ln\left[\frac{VT^{\frac{3}{2}}}{N}\right].$$

We can rewrite *S* in the following way:

(A32) $$S=Ns_{0}+Nk\ln\left[\frac{T^{\frac{3}{2}}V}{N}\right]-\frac{YN^{\frac{3}{2}}}{3V^{\frac{1}{2}}T^{\frac{3}{2}}},$$

where $Y=\frac{\pi^{\frac{1}{2}}e^{3}}{k^{\frac{1}{2}}}$. We know that ${\left[\frac{\partial T}{\partial S}\right]}_{V,N}=\frac{1}{{\left[\frac{\partial S}{\partial T}\right]}_{V,N}}$, then

(A33) $${\left[\frac{\partial S}{\partial T}\right]}_{V,N}=\frac{3}{2}\frac{Nk}{T}+\frac{YN^{\frac{3}{2}}}{2V^{\frac{1}{2}}T^{\frac{5}{2}}}=\frac{1}{2}\left(\frac{3NkV^{\frac{1}{2}}T^{\frac{3}{2}}+YN^{\frac{3}{2}}}{V^{\frac{1}{2}}T^{\frac{5}{2}}}\right).$$

That is,

(A34) $${\left[\frac{\partial^{2}U}{\partial S\partial S}\right]}_{V,N}={\left[\frac{\partial T}{\partial S}\right]}_{V,N}=\frac{1}{{\left[\frac{\partial S}{\partial T}\right]}_{V,N}}.$$

Therefore,

(A35) $${\left[\frac{\partial^{2}U}{\partial S\partial S}\right]}_{V,N}=\left(\frac{2V^{\frac{1}{2}}T^{\frac{5}{2}}}{3NkV^{\frac{1}{2}}T^{\frac{3}{2}}+YN^{\frac{3}{2}}}\right).$$

The other entries of the Hessian are obtained in the same way. They are:

(A36) $${\left[\frac{\partial^{2}U}{\partial V\partial S}\right]}_{N}=-\frac{T}{3V}\left(\frac{6NkV^{\frac{1}{2}}T^{\frac{3}{2}}+YN^{\frac{3}{2}}}{3NkV^{\frac{1}{2}}T^{\frac{3}{2}}+YN^{\frac{3}{2}}}\right),$$

and

(A37) $$\begin{array}{ccc} {\left[\frac{\partial^{2}U}{\partial N\partial S}\right]}_{V} & = & \left(k-s_{0}-k\ln\left[\frac{T^{\frac{3}{2}}V}{N}\right]\right)2\left(\frac{V^{\frac{1}{2}}T^{\frac{5}{2}}}{3NkV^{\frac{1}{2}}T^{\frac{3}{2}}+YN^{\frac{3}{2}}}\right)\\ & & +\left(\frac{YN^{\frac{1}{2}}T}{3NkV^{\frac{1}{2}}T^{\frac{3}{2}}+YN^{\frac{3}{2}}}\right),A37 \end{array}$$

and

(A38) $${\left[\frac{\partial^{2}U}{\partial V\partial V}\right]}_{S,N}=-\frac{1}{2}\left(\frac{N^{\frac{3}{2}}Y-2NkT^{\frac{3}{2}}V^{\frac{1}{2}}}{V^{\frac{5}{2}}T^{\frac{1}{2}}}\right)+\frac{{\left(6NkV^{\frac{1}{2}}T^{\frac{3}{2}}+YN^{\frac{3}{2}}\right)}^{2}}{18V^{\frac{5}{2}}T^{\frac{1}{2}}\left(3NkV^{\frac{1}{2}}T^{\frac{3}{2}}+YN^{\frac{3}{2}}\right)},$$

and

(A39) $$\begin{array}{ccc} {\left[\frac{\partial^{2}U}{\partial N\partial V}\right]}_{S} & = & \left(s_{0}+k\ln\left[\frac{T^{\frac{3}{2}}V}{N}\right]-k\right)\frac{T}{3V}\left(\frac{6NkV^{\frac{1}{2}}T^{\frac{3}{2}}+YN^{\frac{3}{2}}}{3NkV^{\frac{1}{2}}T^{\frac{3}{2}}+YN^{\frac{3}{2}}}\right)\\ & & -\frac{YN^{\frac{1}{2}}}{6V^{\frac{3}{2}}T^{\frac{1}{2}}}\left(\frac{6NkV^{\frac{1}{2}}T^{\frac{3}{2}}+YN^{\frac{3}{2}}}{3NkV^{\frac{1}{2}}T^{\frac{3}{2}}+YN^{\frac{3}{2}}}\right)-\left(\frac{2kV^{\frac{1}{2}}T^{\frac{3}{2}}-YN^{\frac{1}{2}}}{2V^{\frac{3}{2}}T^{\frac{1}{2}}}\right),A39 \end{array}$$

and

(A40) $$\begin{array}{c} \begin{array}{ccc} {\left[\frac{\partial^{2}U}{\partial N\partial N}\right]}_{S,V} & = & \left(-\frac{1}{2}k-s_{0}-k\ln\left[\frac{T^{\frac{3}{2}}V}{N}\right]+\frac{YN^{\frac{1}{2}}+3kV^{\frac{1}{2}}T^{\frac{3}{2}}}{2V^{\frac{1}{2}}T^{\frac{3}{2}}}\right)\\ & & \times \left(k-s_{0}-k\ln\left[\frac{T^{\frac{3}{2}}V}{N}\right]+\frac{YN^{\frac{1}{2}}T}{2V^{\frac{1}{2}}T^{\frac{5}{2}}}\right)\\ & & \times \left(\frac{2V^{\frac{1}{2}}T^{\frac{5}{2}}}{3NkV^{\frac{1}{2}}T^{\frac{3}{2}}+YN^{\frac{3}{2}}}\right)+\frac{2kTV^{\frac{1}{2}}T^{\frac{1}{2}}-YN^{\frac{1}{2}}}{2NV^{\frac{1}{2}}T^{\frac{1}{2}}}. \end{array} \end{array}$$

Note that if we take $S_{corr}=0$, which corresponds to e=0, we obtain the same Hessian matrix of the ideal gas ($H_{pe=0}=H_{ig}$).
